# Territrem and Butyrolactone Derivatives from a Marine-Derived Fungus *Aspergillus* *Terreus*

**DOI:** 10.3390/md12126113

**Published:** 2014-12-17

**Authors:** Xu-Hua Nong, Yi-Fei Wang, Xiao-Yong Zhang, Mu-Ping Zhou, Xin-Ya Xu, Shu-Hua Qi

**Affiliations:** 1 CAS Key Laboratory of Tropical Marine Bio-resources and Ecology, Guangdong Key Laboratory of Marine Materia Medica/RNAM Center for Marine Microbiology, South China Sea Institute of Oceanology, Chinese Academy of Sciences, 164 West Xingang Road, Guangzhou, 510301 Guangdong, China; E-Mails: nongxuhua4883@163.com (X.-H.N.); zhangxiaoyong@scsio.ac.cn (X.-Y.Z.); xuxinya@scsio.ac.cn (X.-Y.X.); 2 Jinan University, 601 West Huangpu Road, Guangzhou, 510632 Guangdong, China; E-Mails: twangyf@jnu.edu.cn (Y.-F.W.); 1208000466@qq.com (M.-P.Z.)

**Keywords:** *Aspergillusterreus*, anti-acetylcholinesterase, anti-HSV-1, antifouling, butyrolactone derivative, territrem derivative

## Abstract

Seventeen lactones including eight territrem derivatives (**1**–**8**) and nine butyrolactone derivatives (**9**–**17**) were isolated from a marine-derived fungus *Aspergillus* *terreus* SCSGAF0162 under solid-state fermentation of rice. Compounds **1**–**3** and **9**–**10** were new, and their structures were elucidated by spectroscopic analysis. The acetylcholinesterase inhibitory activity and antiviral activity of compounds **1**–**17** were evaluated. Among them, compounds **1** and **2** showed strong inhibitory activity against acetylcholinesterase with IC_50_ values of 4.2 ± 0.6, 4.5 ± 0.6 nM, respectively. This is the first time it has been reported that **3**, **6**, **10**, **12** had evident antiviral activity towards HSV-1 with IC_50_ values of 16.4 ± 0.6, 6.34 ± 0.4, 21.8 ± 0.8 and 28.9 ± 0.8 μg·mL^−1^, respectively. Antifouling bioassay tests showed that compounds **1**, **11**, **12**, **15** had potent antifouling activity with EC_50_ values of 12.9 ± 0.5, 22.1 ± 0.8, 7.4 ± 0.6, 16.1 ± 0.6 μg·mL^−1^ toward barnacle *Balanus amphitrite* larvae, respectively.

## 1. Introduction

Alzheimer’s disease (AD) is a neurodegenerative disorder that is the most common cause of dementia among the elderly. Recent studies demonstrated that cholinergic neurodegeneration could be a major pathologic feature of AD [1,2]. Thus, enhancement of the central cholinergic neurotransmission has been regarded as one of the most promising strategies for the symptomatic treatment of AD. Accordingly, acetylcholinesterase (AChE) inhibitors are currently the most effective treatment targets for the design of anti-Alzheimer drug candidates [3].

The arisugacins, territrems and terreulactones, mostly containing α-pyrone and triketide-terpenoid moieties, were isolated from fungi [4,5,6], and most of them showed potent inhibitory activities against AChE [5,6,7,8]. Butyrolactones and aspernolides, with a basal skeleton characterized by a five-membered lactone bearing two aromatic rings [9,10], exhibited a wide range of activities, such as inhibitory activities against AChE [11], β-glucuronidase [12], protein kinase [13], and antimicrobial [11], cytotoxic activities [14].

**Figure 1:**
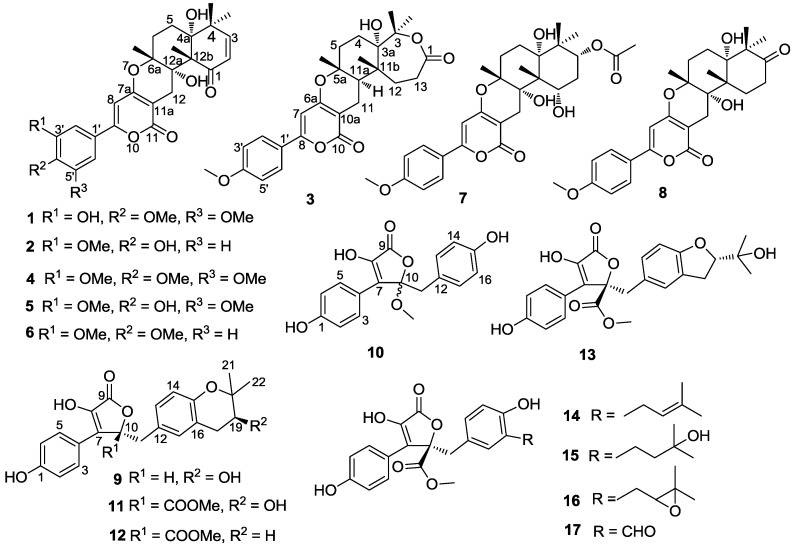
Chemical structures of compounds **1**–**17**.

Recently, we found that the crude extract of the culture medium of a marine-derived fungal strain *Aspergillus* *terreus* SCSGAF0162 had significant AChE inhibitory activity. Further, the bioassay-guided investigation of the extract led to the obtainment of 17 lactones (**1**–**17**) (Figure 1), including eight territrem derivatives and nine butyrolactone derivatives. Among them, compounds **1**–**3** and **9**–**10** were new, and the other compounds were identified as territrem B (**4**) [4], territrem C (**5**) [4], arisugacin A (**6**) [15], arisugacin H (**7**) [5], terreulactone C (**8**) [6], butyrolactone V (**11**) [9,16], aspernolide A (**12**) [10], butyrolactone IV (**13**) [17], butyrolactone I (**14**) [10], aspernolide B (**15**) [10], butyrolactone III (**16**) [11], and 3-hydroxy-4-(4-hydroxyphenyl)-5-methoxycarbonyl-5-(4-hydroxy-3-formylbenzyl)-2,5-dihydro-2-furanone (**17**) [12]. Herein, we report the structural elucidation of new compounds, and the isolation and bioactivity of all the compounds.

## 2. Results and Discussion

**Figure 2:**
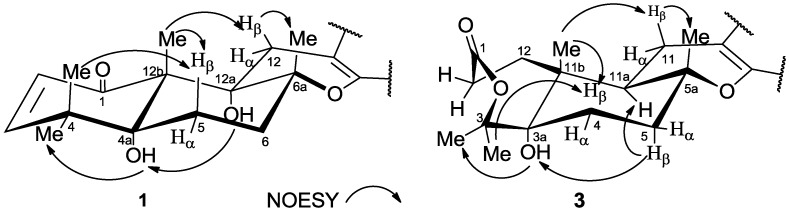
Key NOESY correlations of **1** and **3**.

Compound **1** has a molecular formula of C_28_H_32_O_9_ as determined by HRESIMS (*m/z* 535.1919 [M + Na]^+^). The ^1^H NMR spectrum of **1** showed the presence of six singlet methyl signals and five olefinicmethines. The ^13^C NMR spectrum showed the presence of 28 carbons, including six methyls, three methylenes, five olefinicmethines, and 14 quaternary carbons.These data showed close similarity to those of **6** [15], which suggested that **1** had a territrem skeleton. Comparison of the NMR data of **1** and **6** showed that the only obvious difference between them was the additional appearance of one low-field quaternary carbon (δ_C_ 150.7) and the absence of one aromatic methine in **1**. In the HMBC spectrum, correlations of H-2′ with C-3′/C-4′/C-6′/C-9, H-6′ with C-2′/C-4′/C-5′/C-9, 4′-O*CH_3_* with C-4′, and 5′-O*CH_3_* with C-5′, suggested that C-3′ at the benzene ring was oxygenated. The relative configuration of **1** was confirmed by the NOESY spectrum (Figure 2). NOESY correlations of H-5β with 4β-CH_3_/12b-CH_3_, and H-12β with 6a-CH_3_/12b-CH_3_ suggested that 4β-CH_3_, H-5β, 6a-CH_3_, 12b-CH_3_ and H-12β were in β-oriented, while the NOESY correlations of 4a-OH with 4α-CH_3_/12a-OH indicated 4α-CH_3_, 4a-OH and 12a-OH were in α-oriented. Its relative configuration was identical to that of **6** according to their identical NOESY data. The specific optical rotation value of **1** (${\mathrm{[\alpha ]}}_{\text{D}}^{25}$ +110 (*c* 0.24, CH_3_OH)) was also similar to that of **6** (${\mathrm{[\alpha ]}}_{\text{D}}^{25}$ +144 (*c* 0.10, CHCl_3_)) [15]. These data suggested that the absolute configuration of **1** was the same as that of **6** and determined to be 4a*R*, 6a*R*, 12a*S*, 12b*S*. So, the structure of **1** was elucidated as shown and named territrem D.

Compound **2** has a molecular formula of C_27_H_30_O_8_ as determined by its HRESIMS (*m/z* 505.1822 [M + Na]^+^). Its ^1^H and ^13^C NMR data showed close similarity to those of **6** [15], and the only obvious difference between them was the absence of one oxymethyl group in **2**. The HMBC spectrum showed correlations of H-2′ with C-4′/C-6′/C-9, H-5′ with C-1′/C-3′, H-6′ with C-2′/C-4′/C-9, and 3′-O*CH_3_* with C-3′, suggesting that C-3′ was methyloxygenated and C-4′ was hydroxylated. The relative configuration of **2** was identical to those of **1** and **6** [15] according to their identical NOESY data, which suggested that the absolute configuration of **2** was the same as those of **1** and **6** and assigned to be 4a*R*, 6a*R*, 12a*S*, 12b*S*. So, the structure of **2** was elucidated as shown and named territrem E.

Compound **3** has a molecular formula of C_27_H_32_O_7_ on the basis of its HRESIMS (*m/z* 491.2041 [M + Na]^+^). Its ^1^H and ^13^C NMR data showed similarity to those of **1**, **2**, **6**, and isoterreulactoneA^18^. Comparison of the NMR data of **3** and isoterreulactone A^18^ showed that the obvious difference between them was the lack of one low-field quarterarycarbon (δ_C_ 96.7 in isoterreulactone A) and the addition of one high-field tertiary carbon [δ_C_ 39.9 (CH)] in **3**, which indicated that the only difference between them was that C-11a was not oxygenated in **3**. This was proved by the HMBC spectrum (Figure 2) showing correlations of 5a-CH_3_ with C-5/C-5a/C-11a, and 11b-CH_3_ with C-3a/C-11a/C-11b/C-12. The relative configuration of **3** was determined by the NOESY data (Figure 2). NOESY correlations of H-4β with 3β-CH_3_/11b-CH_3_, and H-11β with 5a-CH_3_/11b-CH_3_, suggested that 3β-CH_3_, H-4β, 5a-CH_3_, 11b-CH_3_ and H-11β were in β-configuration, while the correlations of 3α-CH_3_ with 3a-OH/H-5β, and H-5β with 11a-H, suggested that 3α-CH_3_, 3a-OH, H-5β and 11a-H were in α-configuration. Thus, the relative stereochemistry of C-3a, C-5a, C-11a and C-11b were assigned to be S*, R*, R*, and R*, respectively. Therefore, **3** was named 11a-dehydroxyisoterreulactone A.

Compound **9** has a molecular formula of C_22_H_22_O_6_ as determined by its HRESIMS (*m/z* 405.1310 [M + Na]^+^). Analysis of the ^1^H and ^13^C NMR spectra showed the presence of one 1,4-disubstitued aromatic ring, one 1,4,5-trisubstitued aromatic ring, two methyl groups, one methylene, two oxymethines, one tetra-substituted double bond and one ester carbonyl group. These data showed close similarity to those of **11**^8^, **12**^9^, and **13**^17^, which suggested that **9** also had a butyrolactone skeleton. Comparison of the NMR data of **9** and **11** showed that the only obvious difference between them was the lack of one esterified carboxyl group substituent at C-10 in **9**. This was proved by the HMBC spectrum showing correlations of H-10 with C-7/C-9/C-11/C-12. Thus, the planar structure of **9** was assigned. The coupling constant observed for protons at C-19 and C-18 (*J*_18,19_ = 5.0, 7.5 Hz) suggested that H-19 was equatorial, which demonstrated the configuration of C-19 was *S*. [18] And the similar coupling constant for H-19 in **9** and **11** (*J*_18,19_ = 5.5, 8.4 Hz) further proved the 19*S*-configuration in **9** was as the same as that in **11**. In addition, the β-configuration of H-10 and corresponding 10*R*-configuration of C-10 in **9** was speculated from the proposed biogenetic pathway that **9** was derived from the decarboxylation of **11**, which was further supported by the comparison of CD spectra of **9**, **11** and **12** (see Supplementary Figure S39). In the CD spectra, the experimental data of **9** revealed a good agreement with that of **10** and **11**. So, the structure of **9** was established and named isobutyrolactone V.

Compound **10** has a molecular formula of C_18_H_16_O_6_ as determined by its HRESIMS (*m/z* 351.0842 [M + Na]^+^). Its ^1^H and ^13^C NMR data showed close similarity to those of **14**–**17** [10,11,12,17,19] and butyrolactone II [17], which suggested that **10** had a butyrolactone skeleton. Comparison of the NMR data of **10** and butyrolactone II [17] showed that the obvious difference between them was the substituent at C-10. In the HMBC spectrum, correlations of 10-O*CH_3_* (δ_H_ 3.10, s) with C-10 (δ_C_ 108.3) suggested an oxymethyl group attached at C-10. Compound **10** was inferred as a racemic mixture because its specific rotation was recorded as zero and its CD spectrum did not show a cotton effect. So, **10** was named isobutyrolactone II.

The AChE inhibitory activities of **1**–**17** were evaluated by the modified Ellman method [20]. The results showed that **1**, **2**, **4**–**6**, and **8** exhibited strong inhibiting activity with IC_50_ values of 4.2 ± 0.6, 4.5 ± 0.6, 4.2 ± 0.6, 20.1 ± 3.3, 11.9 ± 2.1, and 50.0 ± 1.5 nM, respectively, compound **7** displayed medium inhibiting activity with IC_50_ value of 5.7 ± 0.8 μM, while other compounds had weak or no activity (see Table 1). The inhibiting activities of **1** and **2** were stronger than the positive control huperzine A (IC_50_ = 39.3 ± 7.6 nM), which indicated that **1** and **2** were new potent AChE inhibitors. The IC_50_ values of **4**, **5**, **6** and **8** were consistent with the literature data [5,21]. The results further proved the conclusion that the enone group at the A-ring was responsible for the AChE inhibition capacity of these territrems, as it involved in binding to the active site of AChE [22,23].

The antiviral activities of **1**–**17** towards HSV-1 were evaluated using plaque reduction assay for the first time. The results showed that under their non-cytotoxic concentrations (TC_0_) against Vero cell line, **3**, **6**, **10**, **12** had evident antiviral activity towards HSV-1 with IC_50_ values of 16.4 ± 0.6, 6.34 ± 0.4, 21.8 ± 0.8 and 28.9 ± 0.8 μg·mL^−1^, respectively, while other compounds did not show clear activity (see Table 1).

Antifouling bioassay tests for **1**–**4** and **8**–**17** showed that compounds **1**, **11**, **12**, **15** had potent antifouling activity at nontoxic concentrations (LC_50_/EC_50_ values > 100 μg·mL^−1^) with EC_50_ values of 12.9 ± 0.5, 22.1 ± 0.8, 7.4 ± 0.6, 16.1 ± 0.6 μg·mL^−1^ towards barnacle *Balanus amphitrite* larvae, respectively, while the other compounds showed weak or no activity (see Table 1). Usually, the standard requirement of an efficacy EC_50_ level for natural antifoulant is 25 μg·mL^−1^, and an antifouling compound with LC_50_/EC_50_ >15 is often considered as a non-toxic antifouling compound [24]. The above data indicate that compounds **1**, **11**, **12**, **15** are potential natural nontoxic antifouling agents.

**Table 1:** The anti-AChE, anti-HSV-1, Cytotoxicity and antifouling activities of **1**–**17**.

Comp.	Anti-AChE IC_50_ (nM)	Anti-HSV-1 IC_50_ (μg·mL^−1^)	Cytotoxicity Against Vero TC_0_ (μg·mL^−1^)	Antifouling Against *B. Amphitrite* EC_50_ (μg·mL^−1^)
**1**	4.2 ± 0.6	NA ^a^	25	12.9 ± 0.5
**2**	4.5 ± 0.6	NA ^a^	200	NA ^a^
**3**	NA ^a^	16.4 ± 0.6	200	NA ^a^
**4**	4.2 ± 0.6	NA ^a^	25	NA ^a^
**5**	20.1 ± 3.3	NA ^a^	>25	NT ^b^
**6**	11.9 ± 2.1	6.34 ± 0.4	100	NT ^b^
**7**	5700 ± 800	NA^a^	100	NT ^b^
**8**	50.0 ± 1.5	NA^a^	>25	NA ^a^
**10**	NA ^a^	21.8 ± 1.8	200	NA ^a^
**11**	NA ^a^	NT ^b^	NT ^b^	22.1 ± 0.8
**12**	NA ^a^	28.9 ± 1.8	100	7.4 ± 0.6
**15**	NA ^a^	NT ^b^	NT ^b^	16.1 ± 0.6
Huperzine A	39.3 ± 7.6	NT ^b^	NT ^b^	NT ^b^
Acyclovir	NT ^b^	34.5 ± 0.7	>1000	NT ^b^

^a^ NA-No activity; ^b^ No test.

## 3. Experimental Section

### 3.1. General Experimental Procedure

Optical rotations were measured with an Anton Paar MCP 500 polarimeter (Anton Paar GmbH, Graz, Austria). UV spectra were obtained using a Shimadzu UV-2600 UV−vis spectrophotometer (Shimadzu, Tokyo, Japan). CD spectra were measured with a Chirascan circular dichroism spectrometer (Applied Photophysics Ltd, London, UK). IR spectra were measured with a Shimadzu IR Affinity-1 Fourier transform infrared spectrophotometer (Shimadzu, Tokyo, Japan). ^1^H, ^13^C NMR and 2D NMR spectra were recorded on a Bruker AV-500 MHz NMR spectrometer (Bruker, Karlsruhe, Germany) with TMS as reference. MS spectroscopic data were obtained on a LCQDECA XP HPLC/MSn spectrometer (Bruker, Karlsruhe, Germany) for ESIMS. High-resolution electrospray-ionization (HRESIMS) was performed on a UPLC/Q-TOF Micro MS spectrometer (Bruker, Karlsruhe, Germany) under 70 eV. Semi-preparative reversed phase (SP-RP) HPLC was performed on a Shimadzu LC-20A preparative liquid chromatography with an YMC-Pack ODS column, 250 × 20 mm i.d., S-5 μm. Sephadex LH-20 (GE Healthcare, London, UK) was used for chromatographic column (CC). Silica gel (200–300 mesh) for CC and GF254 for TLC were obtained from the Qindao Marine Chemical Factory, Qindao, China.

### 3.2. Fungal Material

The fungal strain SCSGAF0162 (GenBank access number JN851044) was isolated from the South China Sea (18°11′ N, 109°25′ E) gorgonian corals *Echinogorgia aurantiaca*, and identified as *Aspergillus terreus* SCSGAF0162 by a molecular biological protocol calling for DNA amplification and ITS region sequence comparison with GenBank database, sharing a similarity of 99% with *Aspergillus niger* EIODSF002 (GenBank access number KJ173525), which was deposited in RNAM center, South China Sea Institute of Oceanology, Chinese Academy of Sciences.

### 3.3. Fermentation and Extraction

The fungal strain was inoculated in PDA liquid medium (containing 20 g/L glucose, 200 g/L potato and 30 g/L sea salt) in 500 mL shake flask loading 120 mL as seed culture and incubated on a rotary shaker (200 rpm) at 28 °C for 3 days. Fermentation of the strain was carried out in 5000 mL Erlenmeyer flasks containing solid-state rice medium (each flask contained 500 g of commercially available rice, yeast extracts 32 g, 24.0 g of sea salt, and 800 mL of water). Then, each of the seed cultures (10 mL) was transferred into autoclaved 5000 mL Erlenmeyer flasks that contained solid-state rice medium. After that, the flasks were incubated at 26 °C as static cultures for 42 days. The total 2 kg of rice culture was crushed and extracted with 80% acetone three times. The acetone extract was evaporated under reduced pressure to afford an aqueous solution, and then the aqueous solution was extracted with EtOAc to yield 30 g of a crude gum.

### 3.4. Purification

The crude extract was subjected to silica gel CC using gradient elution with a CHCl_3_/CH_3_OH solvent system at the ratios of 100:0, 98:2, 95:5, 90:10, 80:20, 50:50, and 0:100 (v/v) to give eight fractions (Fr.1–Fr.8). And the compounds **1**–**17** were isolated and purified by using silica gel CC, MPLC with an ODS column and SP-RP HPLC from the above fractions (more detail see Supplementary Information).

Territrem D (**1**): yellowish amorphous solid; ${\mathrm{[\alpha ]}}_{\text{D}}^{25}$ +110 (*c* 0.24, CH_3_OH); UV (CH_3_OH) λ_max_ (log ε) 328 (4.09), 218 (4.51) nm; IR (CH_3_OH) ν_max_ 3336, 2947, 2831, 1022, cm^−1^; (+)-HRESIMS *m/z* 535.1919 [M + Na]^+^, (calcd for C_28_H_32_NaO_9,_ 535.1939); ^1^H-NMR (500 MHz, DMSO-*d*_6_): δ_H_ 9.53 (1H, br s, 3′-OH), 6.98 (1H, d, *J* = 2.0 Hz, H-2′), 6.97 (1H, d, *J* = 2.0 Hz, H-6′), 6.81 (1H, s, H-8), 6.58 (1H, br s, 12a-OH), 6.35 (1H, d, *J* = 10.0 Hz, H-3), 6.29 (1H, br s, 4a-OH), 5.66 (1H, d, *J* = 10.0 Hz, H-2), 3.83 (3H, s, 5′-O*CH_3_*), 3.71 (3H, s, 4′-O*CH_3_*), 3.49 (1H, d, *J* = 17.5 Hz, H-12α), 2.74 (1H, d, *J* = 17.5 Hz, H-12β), 2.28 (1H, m, H-6 β), 1.96 (1H, m, H-5 β), 1.75 (1H, m, H-5 α), 1.66 (1H, m, H-6 α), 1.39 (3H, s, 6a-CH_3_), 1.36 (3H, s, 12b-CH_3_), 1.20 (3H, s, 4 β*-*CH_3_), 1.07 (3H, s, 4 α-CH_3_); ^13^C-NMR (125 MHz, DMSO-*d*_6_): δ_C_ 200.8 (C, C-1), 163.1 (C, C-11), 162.1 (C, C-7a), 156.7 (C, C-9), 153.4 (C, C-5′), 152.7 (CH, C-3), 150.7 (C, C-3′), 138.2 (C, C-4′), 126.3 (C, C-1′), 123.1 (CH, C-2), 106.3 (CH, C-6′), 100.5 (CH, C-2′), 97.6 (CH, C-8), 97.5 (C, C-11a), 80.6 (C, C-6a), 79.0 (C, C-4a), 74.8 (C, C-12a), 59.8 (CH_3_, 4′-O*CH_3_*), 55.9 (CH_3_, 5′-O*CH_3_*), 55.2 (C, C-12b), 42.0 (C, C-4), 28.3 (CH_2_, C-6), 26.3 (CH_2_, C-12), 25.1 (CH_3_, 4 α-CH_3_), 24.7 (CH_2_, C-5), 23.3 (CH_3_, 6a-CH_3_), 23.2 (CH_3_, 4 β-CH_3_), 21.4 (CH_3_, 12b-CH_3_).

Territrem E (**2**): yellowish amorphous solid; ${\mathrm{[\alpha ]}}_{\text{D}}^{25}$ +129 (*c* 0.33, CH_3_OH); UV (CH_3_OH) λ_max_ (log ε) 337 (3.64), 212 (3.91) nm; IR (CH_3_OH) ν_max_ 3363, 2951, 2839, 1678, 1018 cm^−1^; (+)-HRESIMS *m/z* 505.1822 [M + Na]^+^, (calcd for C_27_H_30_NaO_8_, 505.1806); ^1^H-NMR (500 MHz, DMSO-*d*_6_): δ_H_ 9.73 (1H, br s, 4′-OH), 7.36 (1H, d, *J* = 2.0 Hz, H-2′), 7.32 (1H, dd, *J* = 2.0, 8.5 Hz, H-6′), 6.86 (1H, d, *J* = 8.5 Hz, H-5′), 6.72 (1H, s, H-8), 6.59 (1H, br s, 4a-OH), 6.35 (1H, d, *J* = 10.0 Hz, H-3), 6.29 (1H, br s, 12a-OH), 5.66 (1H, d, *J* = 10.0 Hz, H-2), 3.84 (3H, s, 3′-O*CH_3_*), 3.48 (1H, d, *J* = 17.5 Hz, H-12 α), 2.73 (1H, d, *J* = 17.5 Hz, H-12 β), 2.30 (1H, m, H-6 β), 1.96 (1H, m, H-5 β), 1.72 (1H, m, H-5 α), 1.66 (1H, m, H-6 α), 1.38 (3H, s, 6a-CH_3_), 1.35 (3H, s, 12b-CH_3_), 1.20 (3H, s, 4 β-CH_3_), 1.07 (3H, s, 4 α-CH_3_); ^13^C-NMR (125 MHz, DMSO-*d*_6_): δ_C_ 201.0 (C, C-1), 163.4 (C, C-11), 162.5 (C, C-7a), 157.5 (C, C-9), 152.8 (CH, C-3), 149.2 (C, C-4′), 147.9 (C, C-3′), 123.2 (CH, C-2), 122.4 (C, C-1′), 118.6 (CH, C-6′), 115.7 (CH, C-5′), 108.9 (CH, C-2′), 96.7 (C, C-11a), 96.2 (CH, C-8), 80.6 (C, C-6a), 79.1 (C, C-4a), 75.0 (C, C-12a), 55.7 (CH_3_, 3′-O*CH_3_*), 55.2 (C, C-12b), 42.1 (C, C-4), 28.5 (CH_2_, C-6), 26.4 (CH_2_, C-12), 25.2 (CH_3_, 4 α-CH_3_), 24.8 (CH_2_, C-5), 23.5 (CH_3_, 4 β-CH_3_), 23.3 (CH_3_, 6a-CH_3_), 21.5 (CH_3_, 12b-CH_3_).

12a-dehydroxyisoterreulactone A (**3**): yellowish amorphous solid; ${\mathrm{[\alpha ]}}_{\text{D}}^{25}$ +129 (*c* 0.33, CH_3_OH); UV (CH_3_OH) λ_max_ (log ε) 330 (4.18), 253 (4.14), 206 (4.32) nm; IR (CH_3_OH) ν_max_ 3336, 2943, 2831, 1022 cm^−1^; (+)-HRESIMS *m/z* 491.2041 [M + Na]^+^, (calcd for C_27_H_32_NaO_7_, 491.2040); ^1^H-NMR (500 MHz, DMSO-*d*_6_): δ_H_ 7.82 (2H, d, *J* = 8.5 Hz, H-2′ and H-6′), 7.04 (2H, d, *J* = 8.5 Hz, H-3′ and H-5′), 6.73 (1H, s, H-7), 4.98 (1H, br s, 3a-OH), 3.81 (3H, s, 4′-O*CH_3_*), 2.64 (1H, m, H-12 α), 2.42 (1H, m, H-13 α), 2.34 (1H, m, H-11 α), 2.30 (1H, m, H-13 β), 2.16 (1H, m, H-11 β), 2.02 (1H, m, H-11a), 1.96 (1H, m, H-4 β), 1.87 (2H, m, H-5 α and H-5 β), 1.80 (1H, m, H-4 α), 1.68 (1H, m, H-12 β), 1.33 (3H, s, 3 β-CH_3_), 1.30 (3H, s, 5a-CH_3_), 1.24 (3H, s, 3 α-CH_3_), 1.18 (3H, s, 11b-CH_3_); ^13^C-NMR (125 MHz, DMSO-*d*_6_): δ_C_ 171.5 (C, C-1), 162.8 (C, C-10), 162.4 (C, C-6a), 161.0 (C, C-4′), 157.2 (C, C-8), 126.7 (2CH, C-2′ and C-6′), 123.4 (C, C-1′), 114.3 (2CH, C-3′ and C-5′), 98.1 (C, C-10a), 96.3 (CH, C-7), 89.8 (C, C-3a), 79.5 (C, C-5a), 77.8 (C, C-3), 55.3 (CH_3_, 4′-O*CH_3_*), 39.9 (CH, C-11a), 38.9 (C, C-11b), 33.3 (CH_2_, C-5), 29.4 (CH_3_, 3 α*-*CH_3_), 29.0 (CH_2_, C-12), 28.4 (CH_3_, 3 β-CH_3_), 26.8 (CH_2_, C-13), 26.0 (CH_2_, C-4), 20.5 (CH_3_, 11b-CH_3_), 19.6 (CH_3_, 5a-CH_3_), 16.3 (CH_2_, C-11).

Isobutyrolactone V (**9**): yellowish amorphous solid; ${\mathrm{[\alpha ]}}_{\text{D}}^{25}$ −2 (*c* 0.40, CH_3_OH), UV (CH_3_OH) λ_max_ (log ε) 302 (4.10), 220 (4.02), 205 (4.27) nm; CD (2.618 mM, CH_3_OH) λ_max_ 230 (−0.22), 258 (0.49), 288 (−1.22), 352 (0.29); IR (CH_3_OH) ν_max_ 3372, 1732, 1666, 1609, 1204, 1146 cm^−1^; (+)-HRESIMS *m/z* 405.1310 [M + Na]^+^, (calcd for C_22_H_22_NaO_6_, 405.1309). ^1^H-NMR (500 MHz, CD_3_OD): δ_H_ 7.60 (2H, dd, *J* = 2.0, 8.5 Hz, H-3 and H-5), 6.92 (2H, dd, *J* = 2.0, 8.5 Hz, H-2 and H-6), 6.73 (1H, dd, *J* = 1.5, 8.0 Hz, H-13), 6.67 (1H, brs, H-17), 6.59 (1H, d, *J* = 8.0 Hz, H-14), 5.61 (1H, dd, *J* = 3.5, 5.5 Hz, H-10), 3.74 (1H, dd, *J* = 5.0, 7.5 Hz, H-19), 3.26 (1H, dd, *J* = 3.5, 14.5 Hz, H-11a), 2.91 (1H, m, H-11b), 2.89 (1H, dd, *J* = 5.0, 16.0 Hz, H-18a), 2.65 (1H, dd, *J* = 7.5, 16.0 Hz, H-18b), 1.31 (3H, s, CH_3_-21), 1.22 (3H, s, CH_3_-22); ^13^C-NMR (125 MHz, CD_3_OD): δ_C_ 171.8 (C, C-9), 159.4 (C, C-1), 153.3 (C, C-15), 137.9 (C, C-8), 132.3 (CH, C-17), 130.4 (2CH, C-3 and C-5), 129.9 (CH, C-13), 129.8 (C, C-12), 128.0 (C, C-4), 123.7 (C, C-7), 120.8 (C, C-16), 117.5 (CH, C-14), 116.7 (2CH, C-2 and C-6), 80.4 (CH, C-10), 78.0 (C, C-20), 70.6 (CH, C-19), 39.8 (CH_2_, C-11), 32.2 (CH_2_, C-18), 25.9 (CH_3_, C-21), 20.9 (CH_3_, C-22).

Isobutyrolactone II (**10**): yellowish amorphous solid; ${\mathrm{[\alpha ]}}_{\text{D}}^{25}$ −3 (*c* 1.4, CH_3_OH), UV (CH_3_OH) λ_max_ (log ε) 309 (4.31), 223 (4.20), 204 (4.26) nm, IR (CH_3_OH) ν_max_ 3367, 1748, 1609, 1516 cm^−1^; (+)-HRESIMS *m/z* 351.0842 [M + Na]^+^, (calcd for C_18_H_16_NaO_6_, 351.0839); ^1^H-NMR (500 MHz, DMSO-*d*_6_): δ_H_ 7.73 (2H, d, *J* = 8.5 Hz, H-3 and H-5), 6.91 (2H, d, *J* = 8.5 Hz, H-2 and H-6), 6.63 (2H, d, *J* = 8.0 Hz, H-13 and H-17), 6.53 (2H, d, *J* = 8.0 Hz, H-14 and H-16), 3.20 (1H, d, *J* = 14.0 Hz, H-11b), 3.15 (1H, d, *J* = 14.0 Hz, H-11a), 3.10 (3H, s, 10-O*CH_3_*); ^13^C-NMR (125 MHz, DMSO-*d*_6_): δ_C_ 166.2 (C, C-9), 157.8 (C, C-1), 156.1 (C, C-15), 139.1 (C, C-8), 131.1 (2CH, C-13 and C-17), 128.9 (2CH, C-3 and C-5), 123.9 (C, C-12), 123.2 (C, C-7), 121.1 (C, C-4), 115.8 (2CH, C-2 and C-6), 114.5 (2CH, C-14 and C-16), 108.3 (CH, C-10), 50.0 (CH_3_, 10-O*CH_3_*), 42.3 (CH_2_, C-11).

### 3.5. Enzyme-Based Assay of AChE

The inhibitory activities against AChE of compounds were investigated *in vitro* using the modified Ellman method [20]. Briefly, the reaction mixture containing 20 μL of a different concentrations of the test compunds dissolved in DMSO solution, 450 μL of reaction buffer (0.01 M phosphate buffer, pH 7.0), 10 μL of 0.08-0.10 units/mL AChE (Sigma, one unit hydrolyzes 1.0 mmol of acetylcholineto choline and acetate per min at pH 7.0, 37 °C) and 10 μL of 0.01 M DTNB (Sigma, St. Louis, MO, USA) were filled in a microwell of 24-well polystyrene plate and incubated for 30 min at 37 °C. After preincubation, The reaction was started by adding 20 μL of 0.01 M ATCh (Sigma, St. Louis, MO, USA) solution in 0.01 M phosphate buffer (pH 7.0). Colorimetric measurements (412 nm) were performed on a enspire multimode microplate reader (varioskan flash, Thermo, Waltham, MA, USA). For determining the blank value, the 20 μL of test compound solution was instead of 20 μL of DMSO solution. Each concentration was analyzed in triplicate. The inhibition of the enzyme was calculated from the slope of the linear part of the enzyme reaction (absorption vs time) in relation to controls (no inhibition, 100% activity). IC_50_ values were determined graphically from the regression analysis of concentration-inhibition curves. The IC_50_ values are the mean ± standard deviations of three independent experiments. The inhibitory effects are represented as compounds concd (nM) giving 50% inhibition on AChE activity (IC_50_).

### 3.6. Plaque Reduction Assay

Cytotoxic activity was evaluated using Vero cell lines by the MTT method. Anti-HSV-1 activity was determined by plaque assay using monolayer cultures of Vero cells in 24-well culture plates (Corning, New York, NY, USA). Virus suspension containing HSV-1 (30 plaque forming units (PFU)/well) was added to the cell wells and incubated at 37 °C with 5% CO_2_ for 2 h. The virus inoculum was then removed and overlay medium (maintenance medium containing 1% methylcellulose and various concentrations of tested compound) was added to each well. After another 72 h of incubation, the cell monolayers were fixed with 10% formalin and stained with 1% crystal violet. Plaques were counted and the percentage of inhibition was calculated according to literature [25]. The concentration reducing plaque numbers by 50% was calculated by regression analysis of the dose–response curves generated from the plaque assay and was defined as 50% inhibitory concentration (IC_50_).

### 3.7. Barnacle Balanus Amphitrite Larval Settlement Bioassays

Larval settlement bioassays were performed using sterile 24-well polystyrene plates. Tested samples were dissolved in DMSO to a concentration of 50 μg·mL^−1^ for preliminary bioassay. To define the EC_50_ values of anti-larval compounds found in the preliminary bioassay, different dilutions of the tested compounds were further prepared to the concentrations ranging from 0.1 to 200 μg·mL^−1^ in autoclaved FSW. About 20 competent larvae were added to each well in 1 mL of the test solution. The experiment was repeated twice with four replicateseach time. Wells containing only FSW with DMSO served as the controls. The plates were incubated at 27 °C for 24 h. The percentage of larval settlement was determined by counting the settled, live individuals under a dissecting microscope and expressing the result as a proportion of the total number of larvae in the well. Statistical calculations were performed with the SPSS software package. EC_50_ (inhibits 50% of settlement of *B. amphitrite* larvae in comparison with the control) levels of tested compounds were calculated by using the Probit software program [26].

## 4. Conclusions

In this study, 17 lactones including eight territrem derivatives (**1**–**8**) and nine butyrolactone derivatives (**9**–**17**) were isolated from a marine-derived fungus *Aspergillus* *terreus* SCSGAF0162. Compounds **1**–**3** and **9**–**10** were new, which extended the territrem and butyrolactone family by derivatives. Among these compounds, **1**, **2**, **4**–**6**, and **8** showed strong inhibiting activity against acetylcholinesterase, and **7** showed medium inhibiting activity against acetylcholinesterase. Until now, compounds **1**, **2**, and **4** were the strongest AChE inhibitors of the territrem family. In addition, **3**, **6**, **10**, and **12** were found to have obvious antiviral activity towards HSV-1 for the first time. Furthermore, **1**, **11**, **12**, and **15** were found to have potent antifouling activity with non- or low toxicity. This study also expanded the bioactivity of the territrem and butyrolactone families.

## Supplementary Files

Supplementary File 1
