# Supplementary material for: Territrem and Butyrolactone Derivatives from a Marine-Derived Fungus Aspergillus Terreus
Source: Mar Drugs. 2014 Dec 17;12(12):6113–24. doi: 10.3390/md12126113 (PMC4278221; doi:10.3390/md12126113)
Supplement: Supplementary File 1 [file marinedrugs-12-06113-s001.pdf]

Supplementary Information

Figure S1. ^{1}H NMR spectrum of **1** S2
Figure S2. Amplifying ^{1}H NMR spectrum of **1** S2
Figure S3. ^{13}C NMR spectrum of **1** S3
Figure S4. DEPT135 spectrum of **1** S3
Figure S5. HSQC spectrum of **1** S4
Figure S6. HMBC spectrum of **1** S4
Figure S7. NOESY spectrum of **1** S5
Figure S8. Amplifying NOESY spectrum of **1** S5
Figure S9. HRESIMS spectrum of **1** S6
Figure S10. IR spectrum of **1** S6
Figure S11. ^{1}H NMR spectrum of **2** S7
Figure S12. ^{13}C NMR and DEPT135 spectra of **2** S7
Figure S13. HSQC spectrum of **2** S8
Figure S14. HMBC spectrum of **2** S8
Figure S15. H-H COSY spectrum of **2** S9
Figure S16. NOESY spectrum of **2** S9
Figure S17. HRESIMS spectrum of **2** S10
Figure S18. IR spectrum of **2** S10
Figure S19. ^{1}H NMR spectrum of **3** S11
Figure S20. ^{13}C NMR and DEPT135 spectra of **3** S11
Figure S21. HSQC spectrum of **3** S12
Figure S22. HMBC spectrum of **3** S12
Figure S23. H-H COSY spectrum of **3** S13
Figure S24. NOESY spectrum of **3** S13
Figure S25. HRESIMS spectrum of **3** S14
Figure S26. IR spectrum of **3** S14
Figure S27. ^{1}H NMR spectrum of **9** S15
Figure S28. ^{13}C NMR spectrum of **9** S15
Figure S29. HSQC spectrum of **9** S16
Figure S30. HRESIMS spectrum of **9** S16
Figure S31. IR spectrum of **9** S17
Figure S32. ^{1}H NMR spectrum of **10** S17
Figure S33. ^{13}C NMR and DEPT135 spectra of **10** S18
Figure S34. HSQC spectrum of **10** S18
Figure S35. HMBC spectrum of **10** S19
Figure S36. H-H COSY spectrum of **10** S19
Figure S37. HRESIMS spectrum of **10** S20
Figure S38. IR spectrum of **10** S20
Figure S39. Comparison of the experimental CD spectra of **9**, **11**, **12** S21
S1. Isolation and purification of compounds **1–17** S21
S2. Structures of compounds **1–17** S22

Figure S1. ^{1}H-NMR spectrum of **1**.

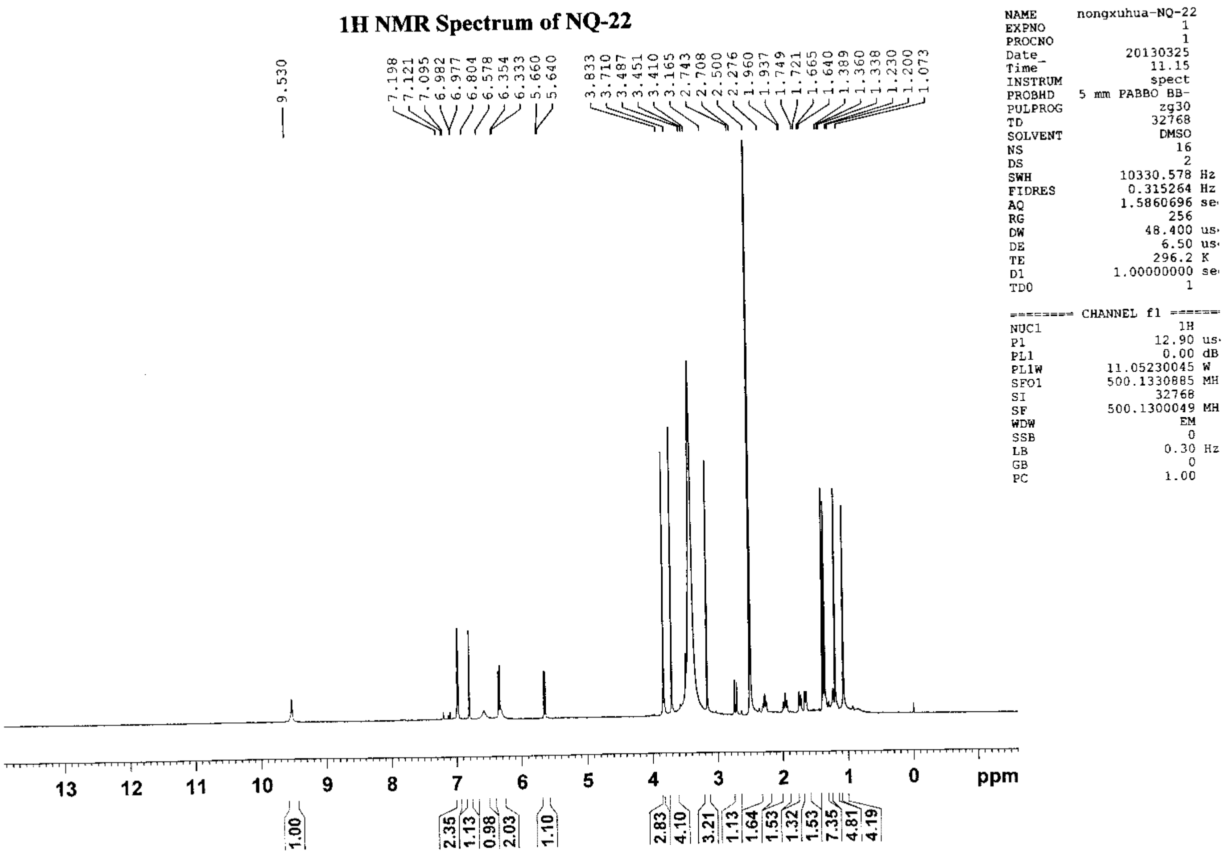

Figure S2. Amplifying ^{1}H-NMR spectrum of **1**.

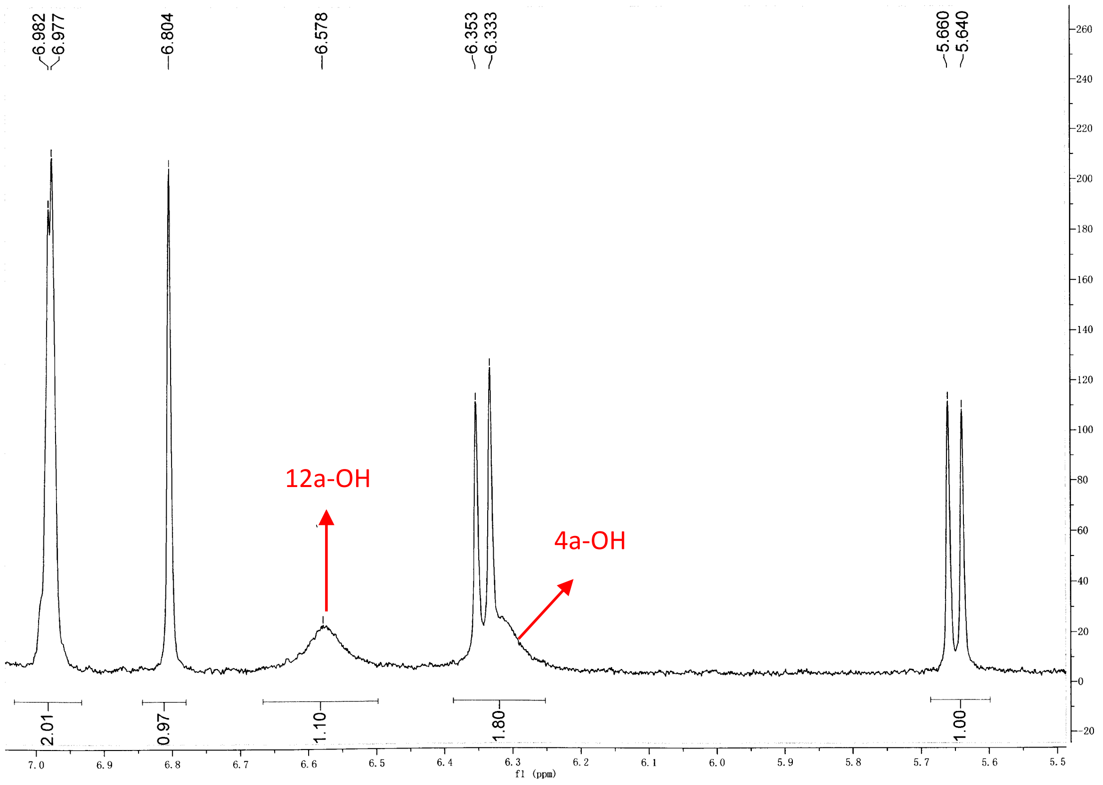

Figure S3. ^{13}C-NMR spectrum of **1**.

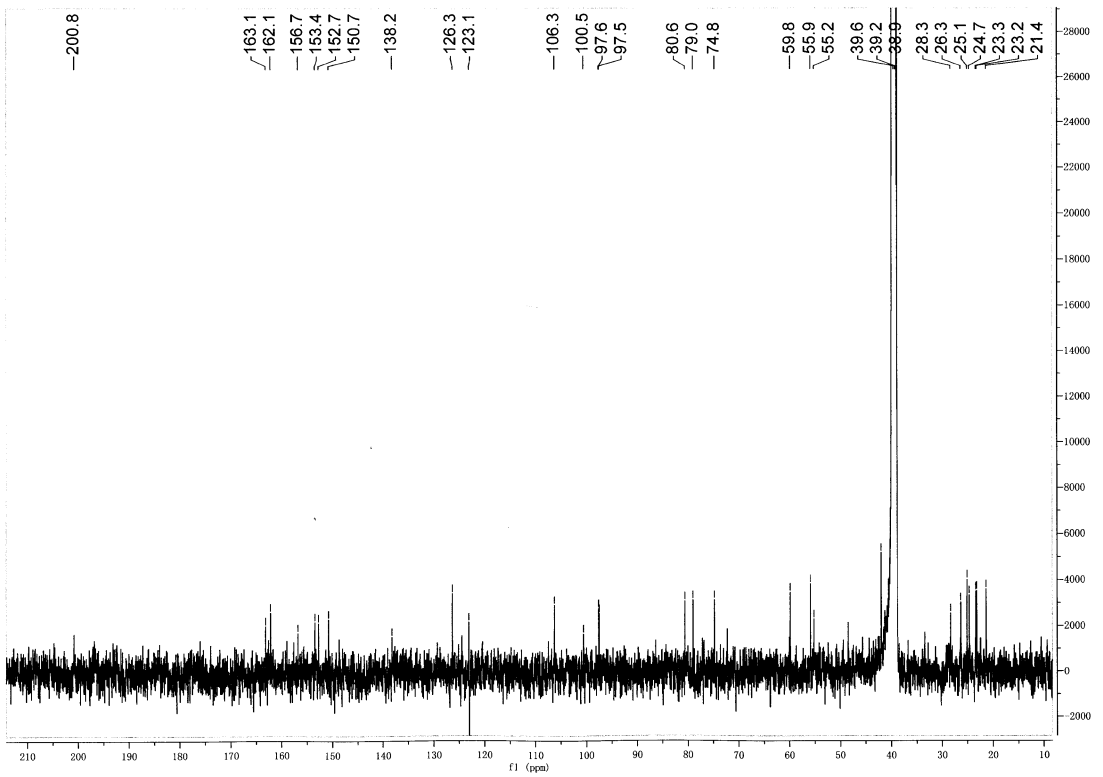

Figure S4. DEPT135 spectrum of **1**.

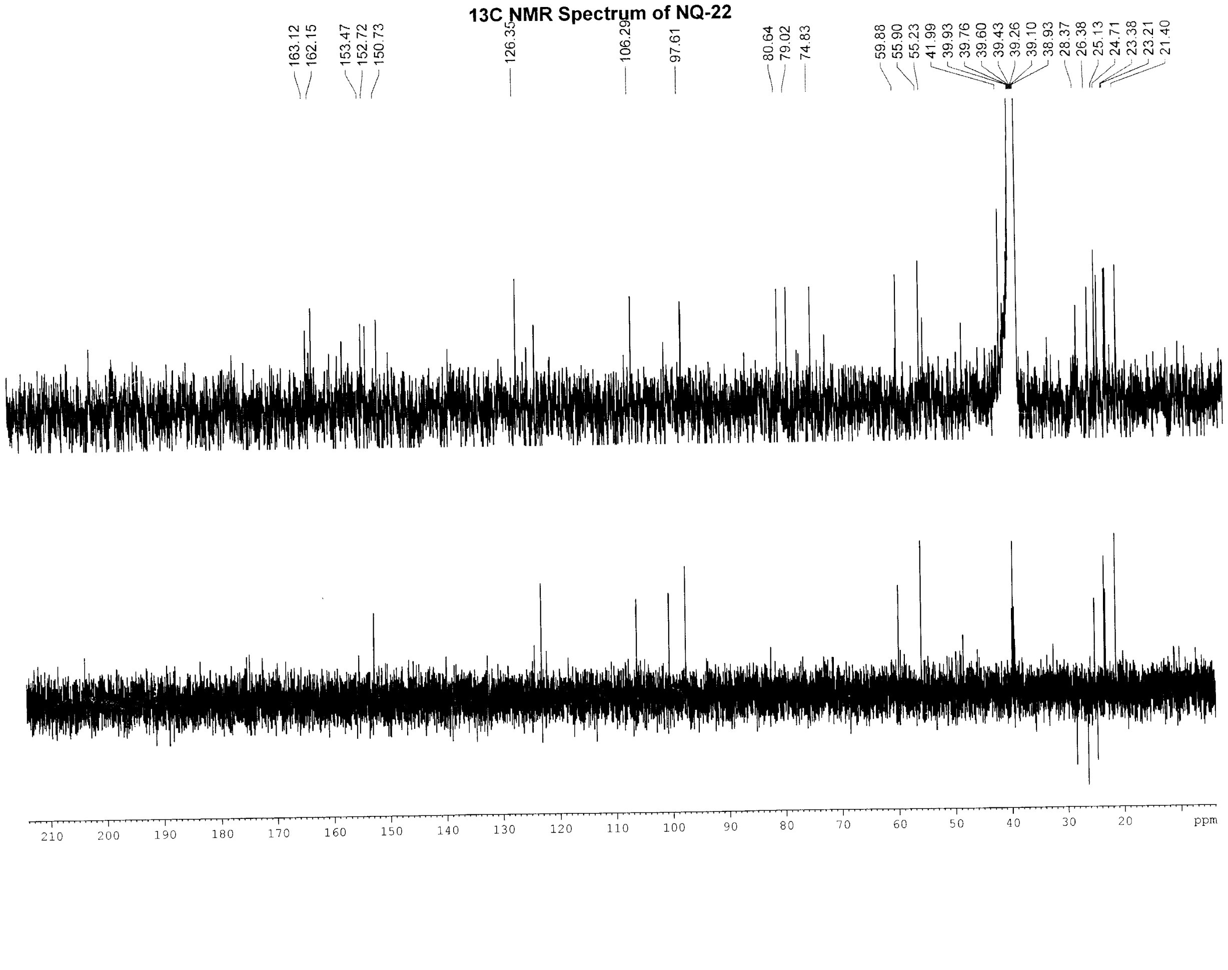

Figure S5. HSQC spectrum of **1**.

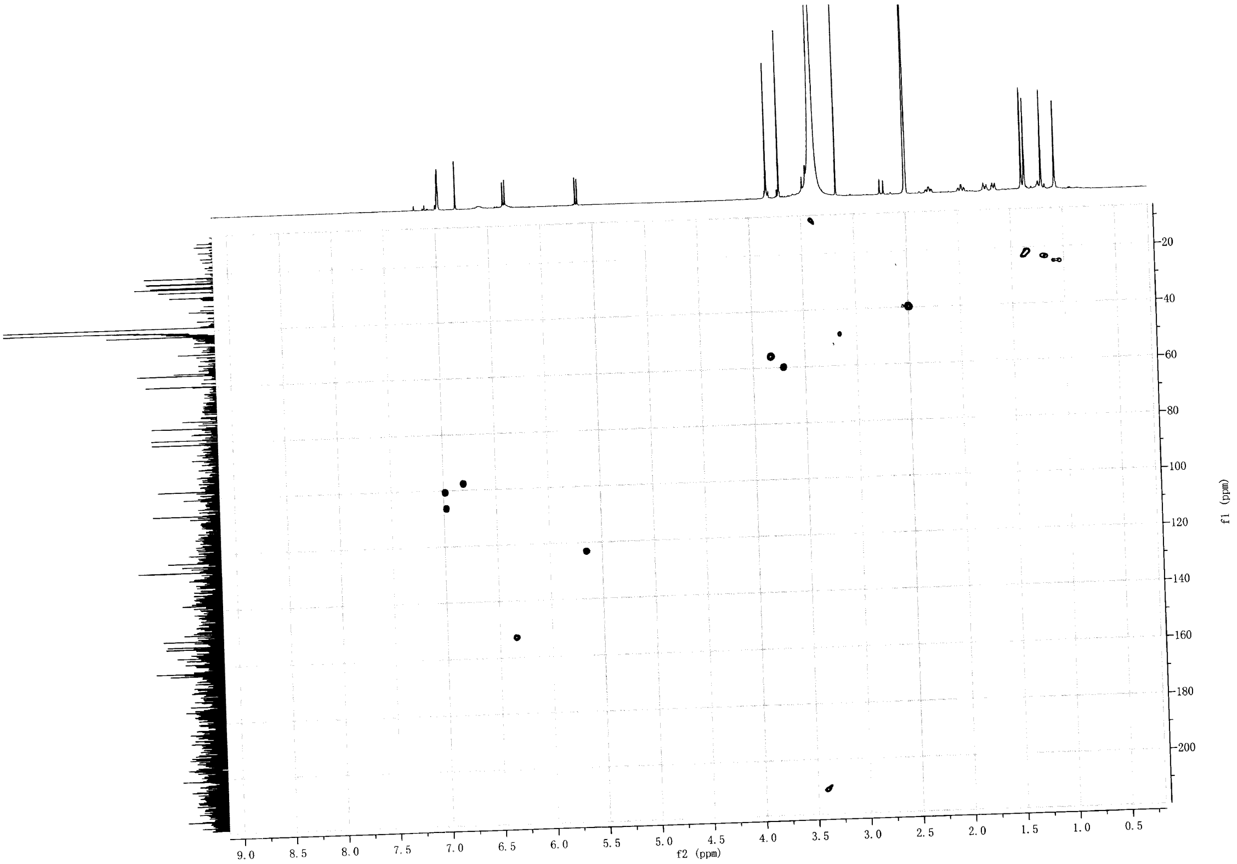

Figure S6. HMBC spectrum of **1**.

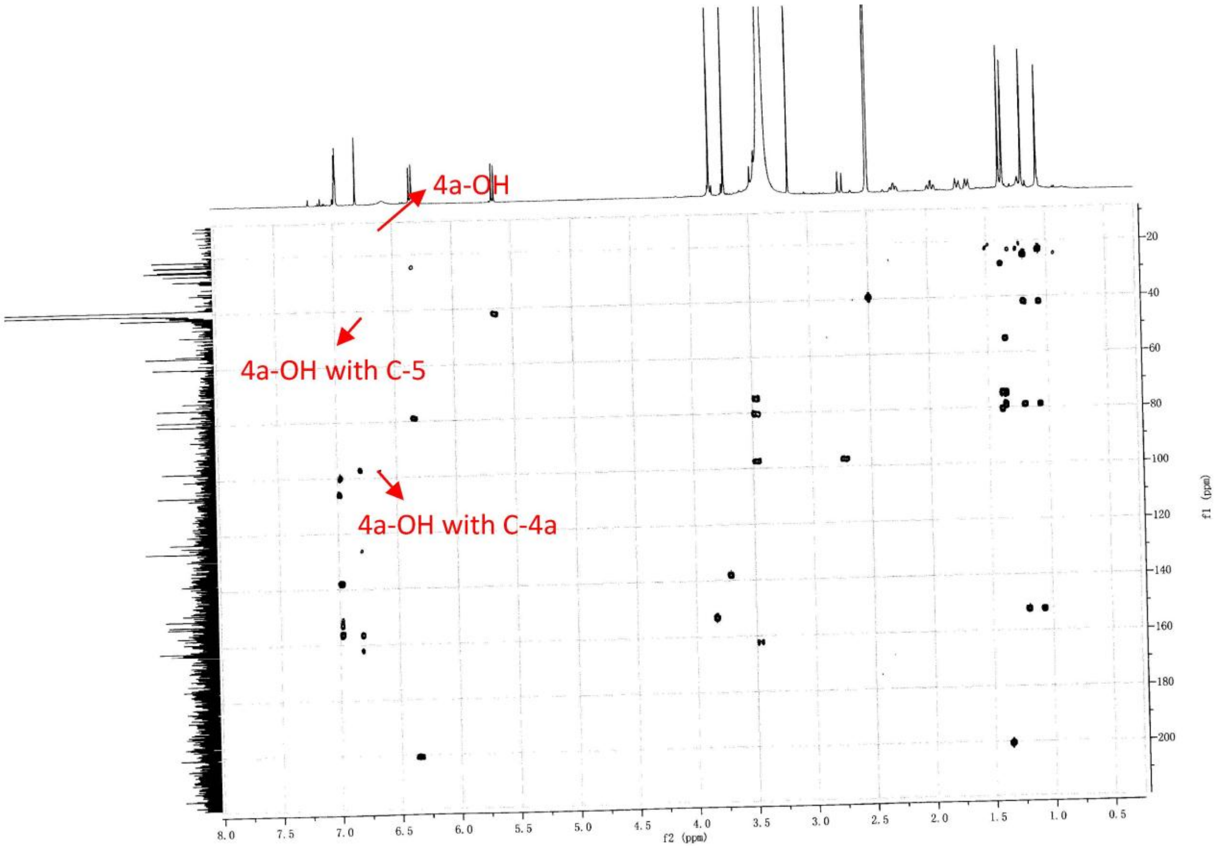

Figure S7. NOE spectrum of **1**.

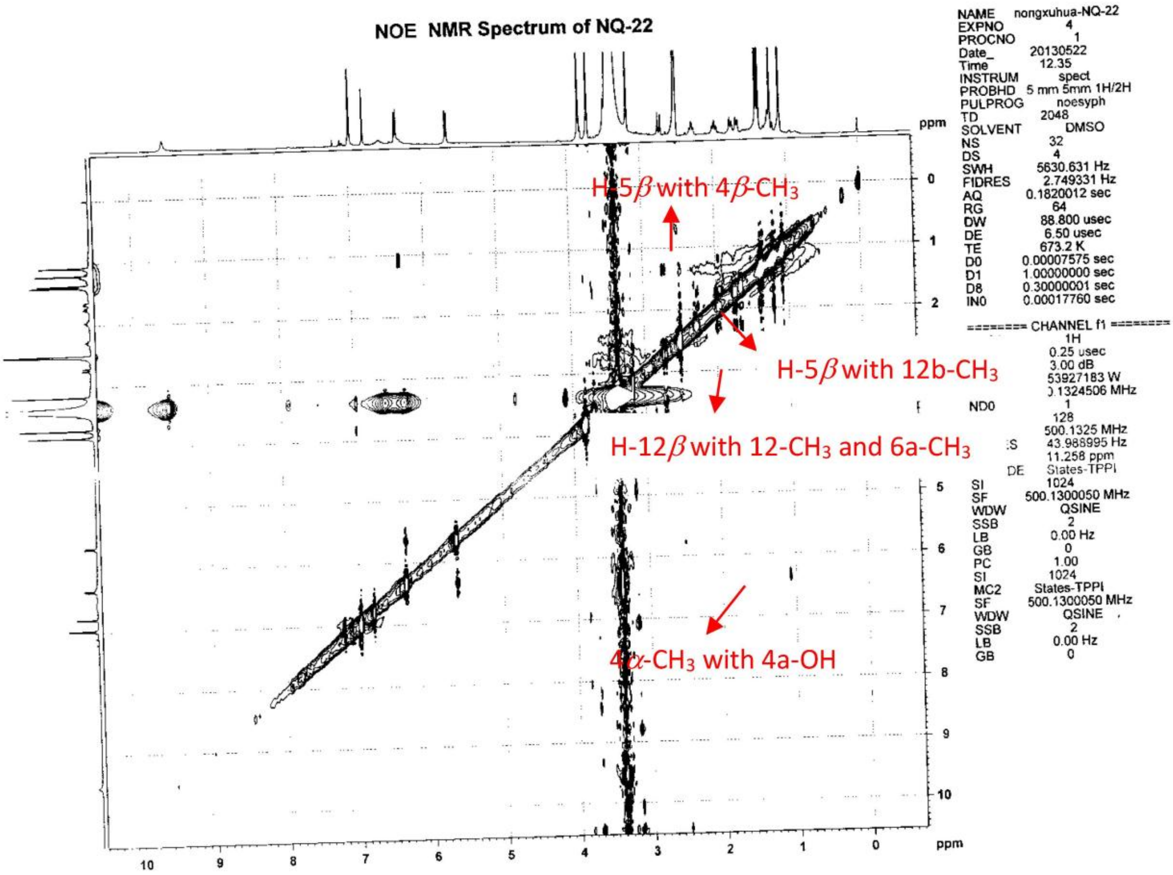

Figure S8. Amplifying NOE spectrum of **1**.

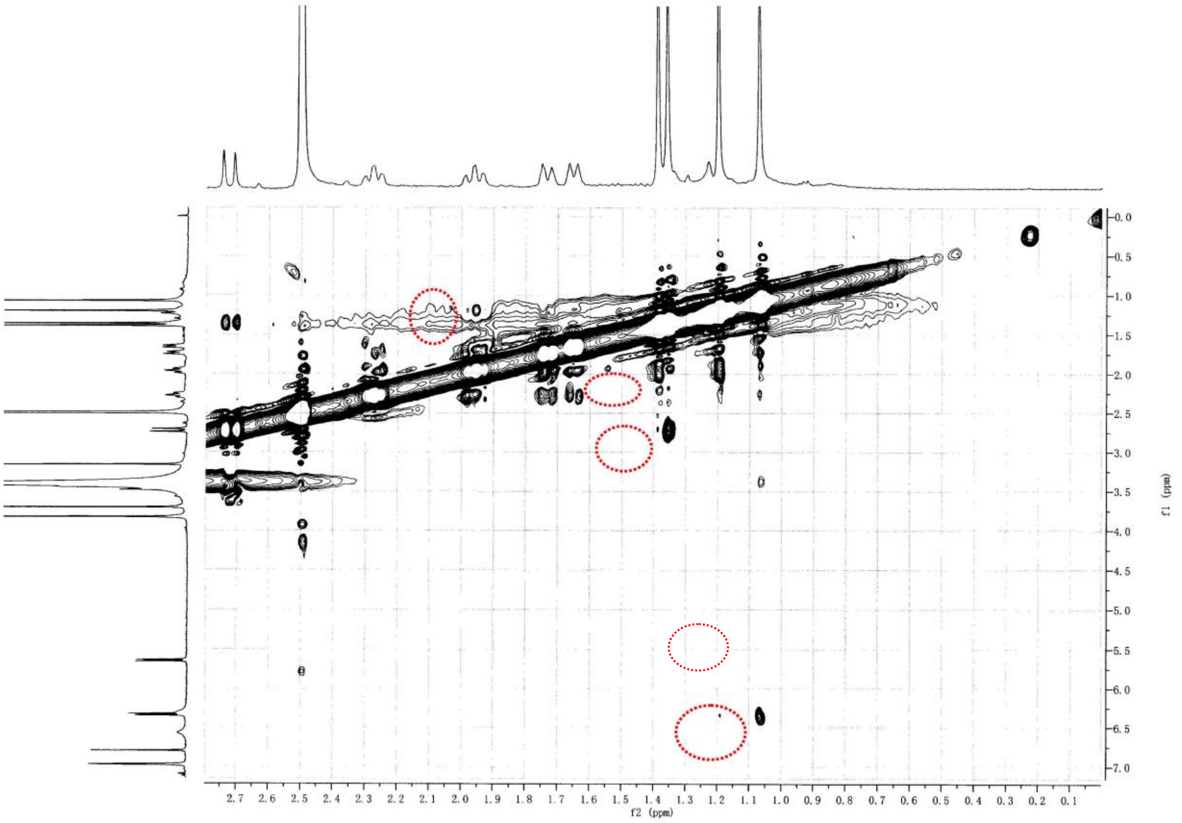

Figure S9. HR-ESIMS spectrum of **1**.

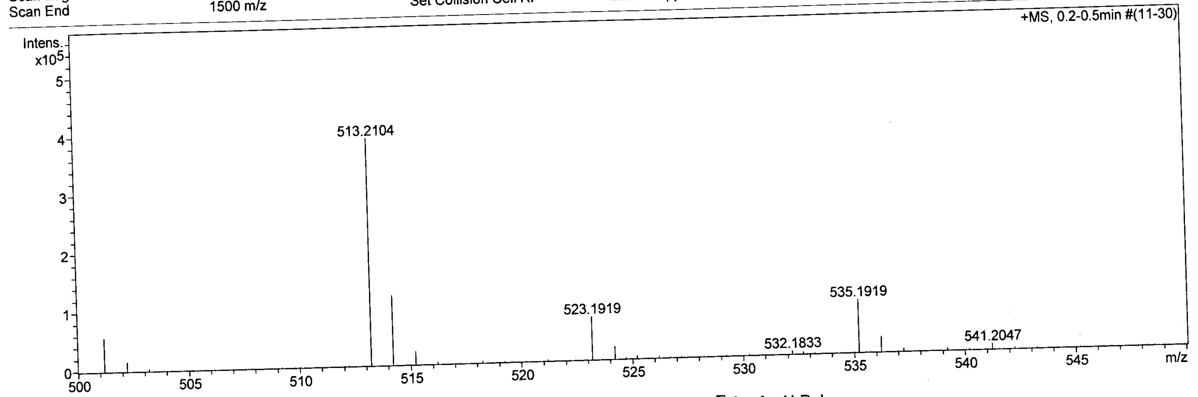

Mass Spectrum SmartFormula Report

Analysis Info

Analysis Name	D:\Data\MS\data\201311\nongxuhua_NQ-22_pos.d	Acquisition Date	11/26/2013 2:58:29 PM
Method	POS_100-2000_Dirrect Infusion.m	Operator	SCSIO
Sample Name	SCSIO	Instrument / Ser#	maXis 29
Comment			

Acquisition Parameter

Source Type	ESI	Ion Polarity	Positive	Set Nebulizer	0.3 Bar
Focus	Not active	Set Capillary	4500 V	Set Dry Heater	180 °C
Scan Begin	100 m/z	Set End Plate Offset	-500 V	Set Dry Gas	4.0 l/min
Scan End	1500 m/z	Set Collision Cell RF	2000.0 Vpp	Set Divert Valve	Waste

Meas. m/z	#	Formula	Score	m/z	err [mDa]	err [ppm]	mSigma	rdb	e⁻ Conf	N-Rule
513.2104	1	C 28 H 33 O 9	100.00	513.2119	1.5	3.0	3.4	12.5	even	ok
535.1919	1	C 28 H 32 Na O 9	100.00	535.1939	2.0	3.7	8.1	12.5	even	ok
1025.4123	1	C 56 H 65 O 18	100.00	1025.4165	4.3	4.2	18.3	24.5	even	ok

Bruker Compass DataAnalysis 4.0 printed: 11/26/2013 3:20:37 PM Page 1 of 1

Figure S10. IR spectrum of **1**.

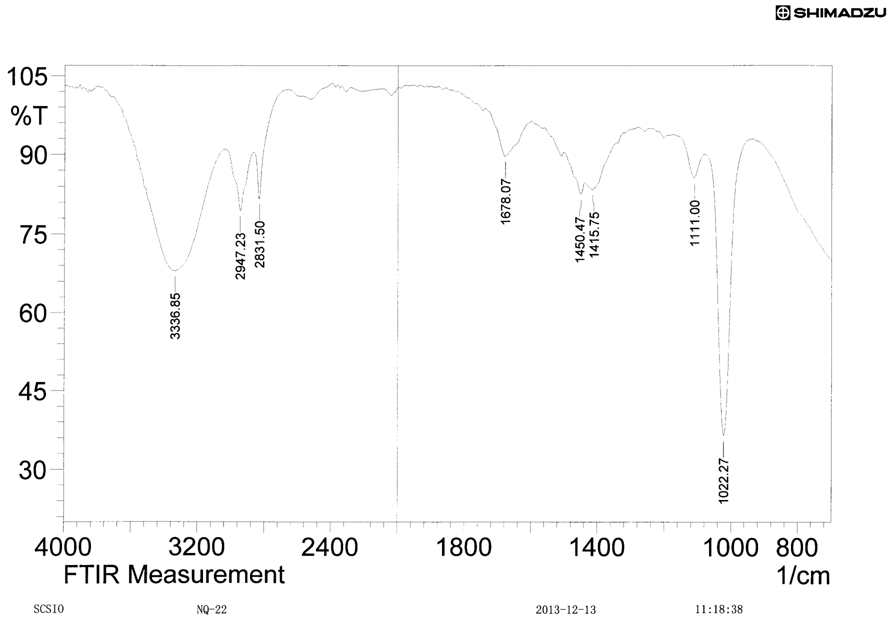

Figure S11. ^{1}H-NMR spectrum of **2**.

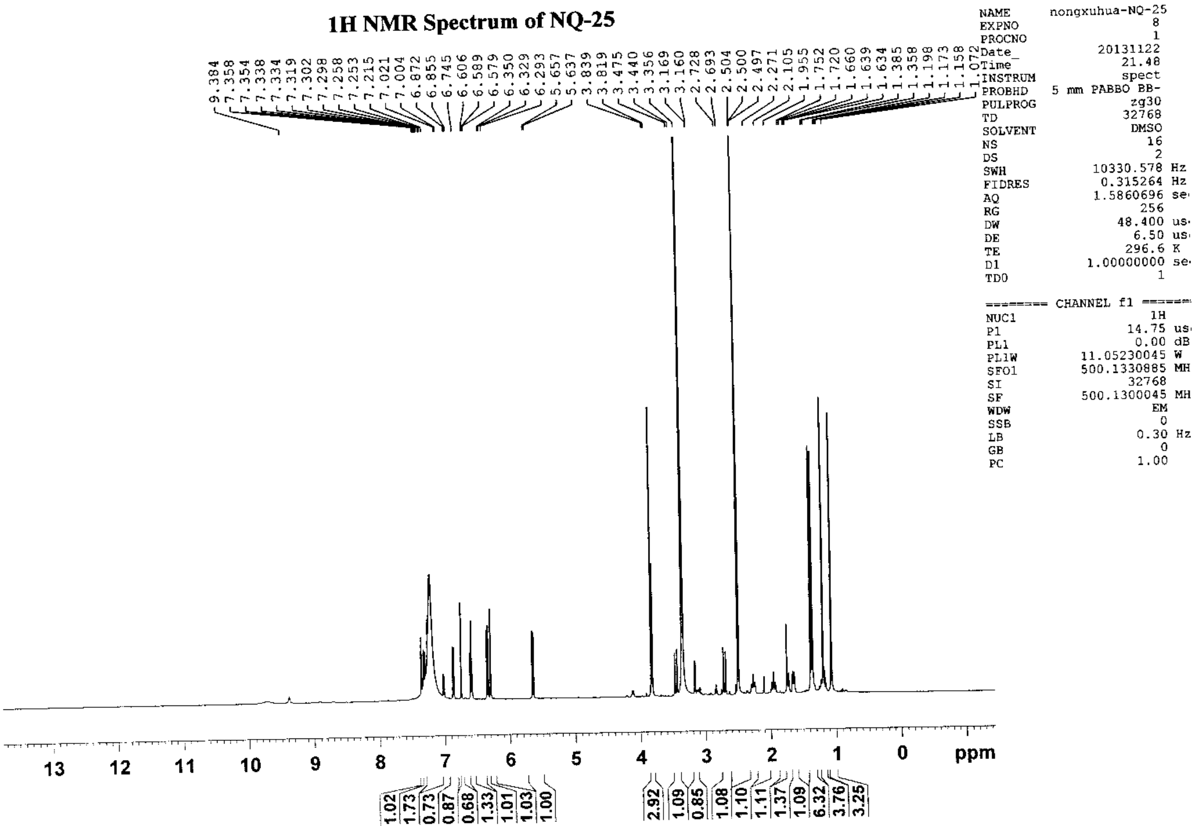

Figure S12. ^{13}C-NMR and *DEPT135* spectra of **2**.

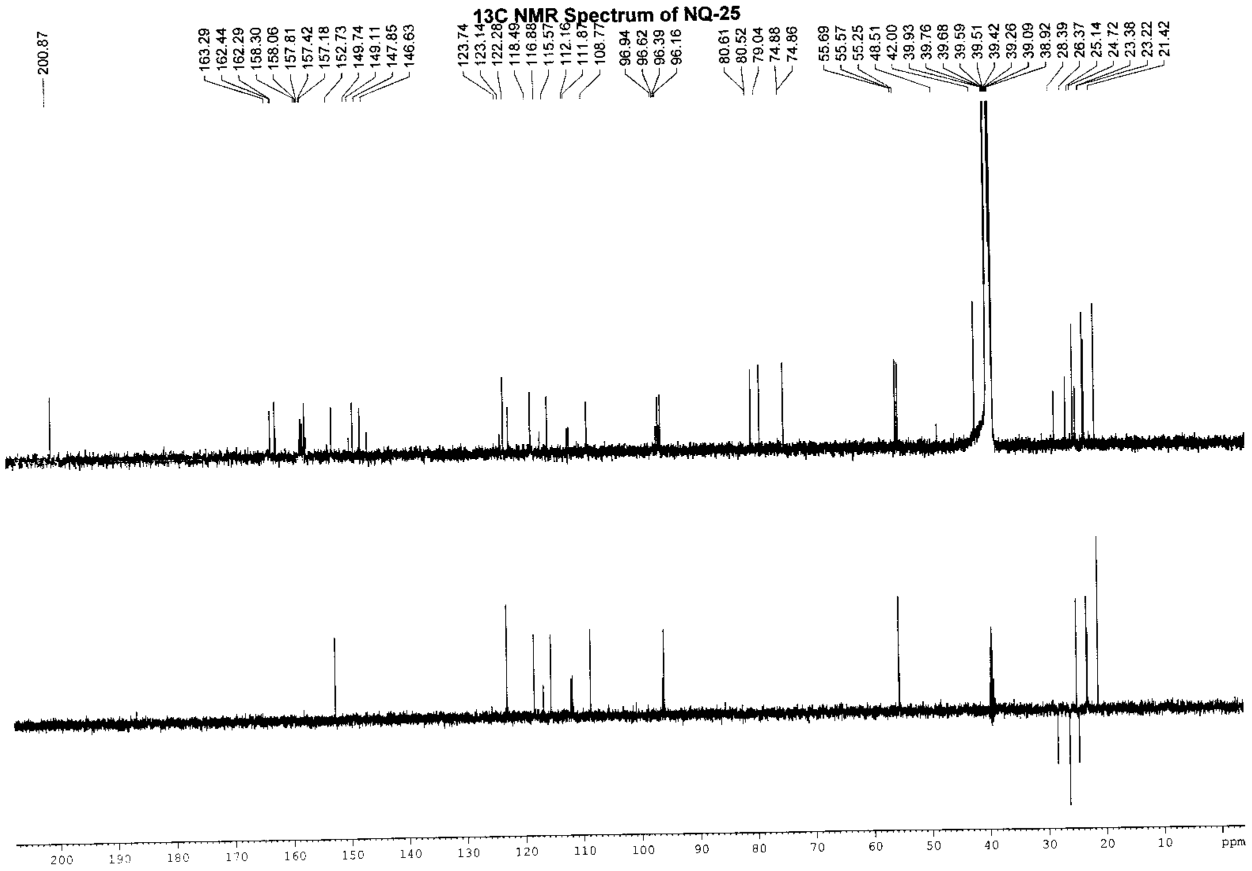

Figure S13. HSQC spectrum of **2**.

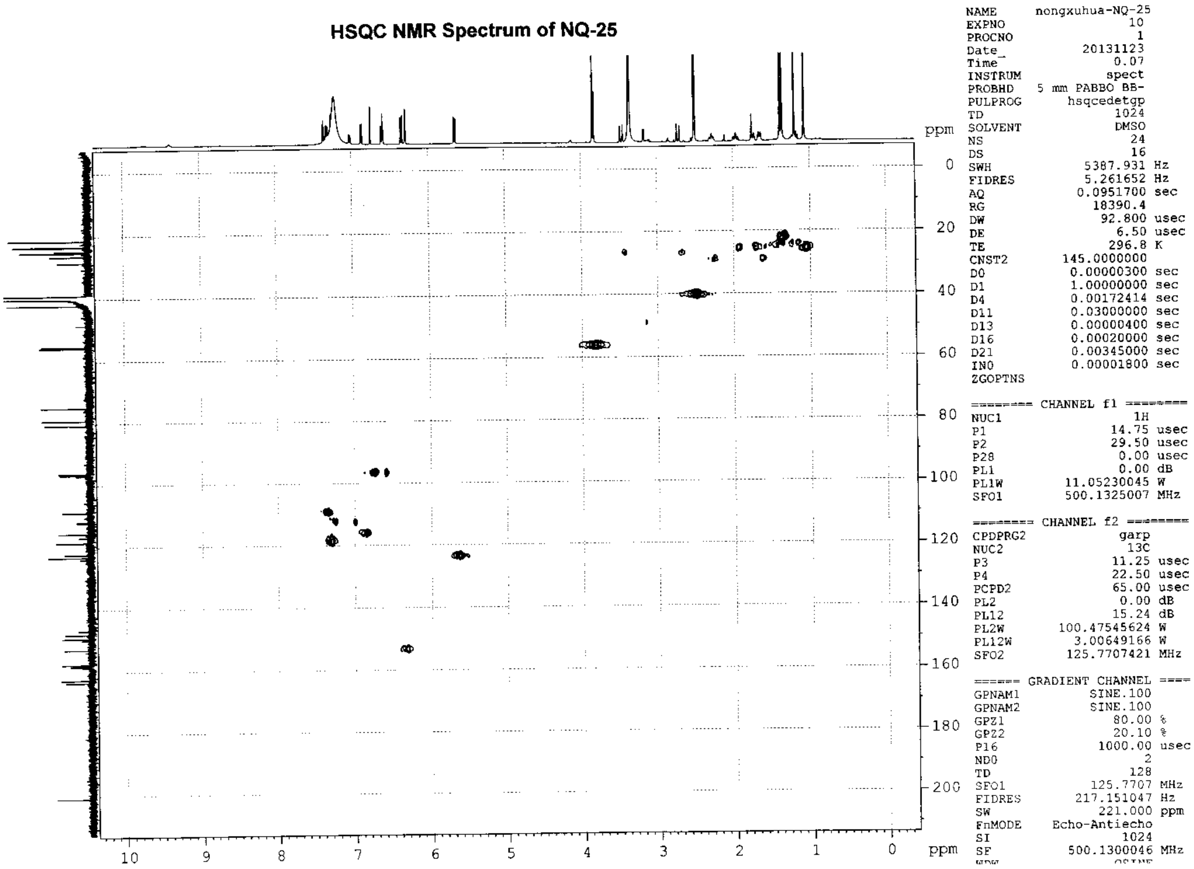

Figure S14. HMBC spectrum of **2**.

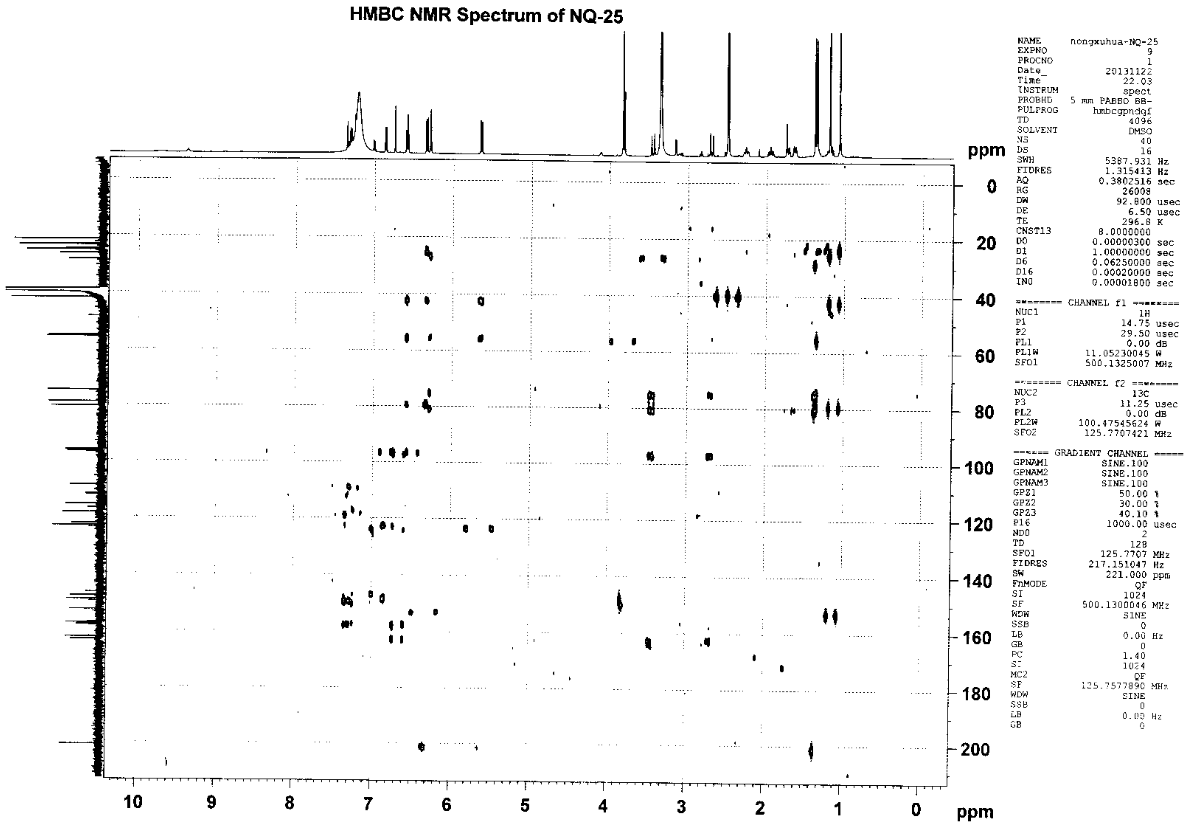

Figure S15. ^{1}H-^{1}H COSY spectrum of **2**.

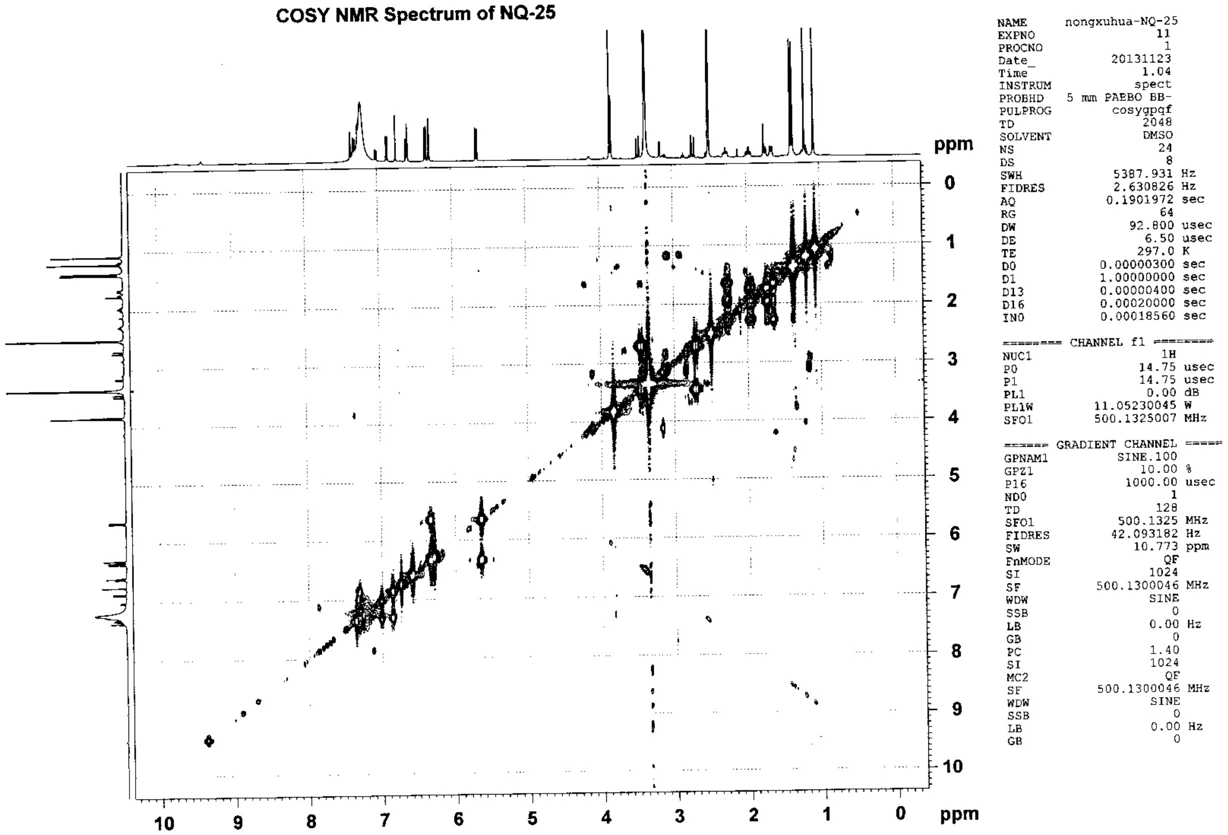

Figure S16. NOE spectrum of **2**.

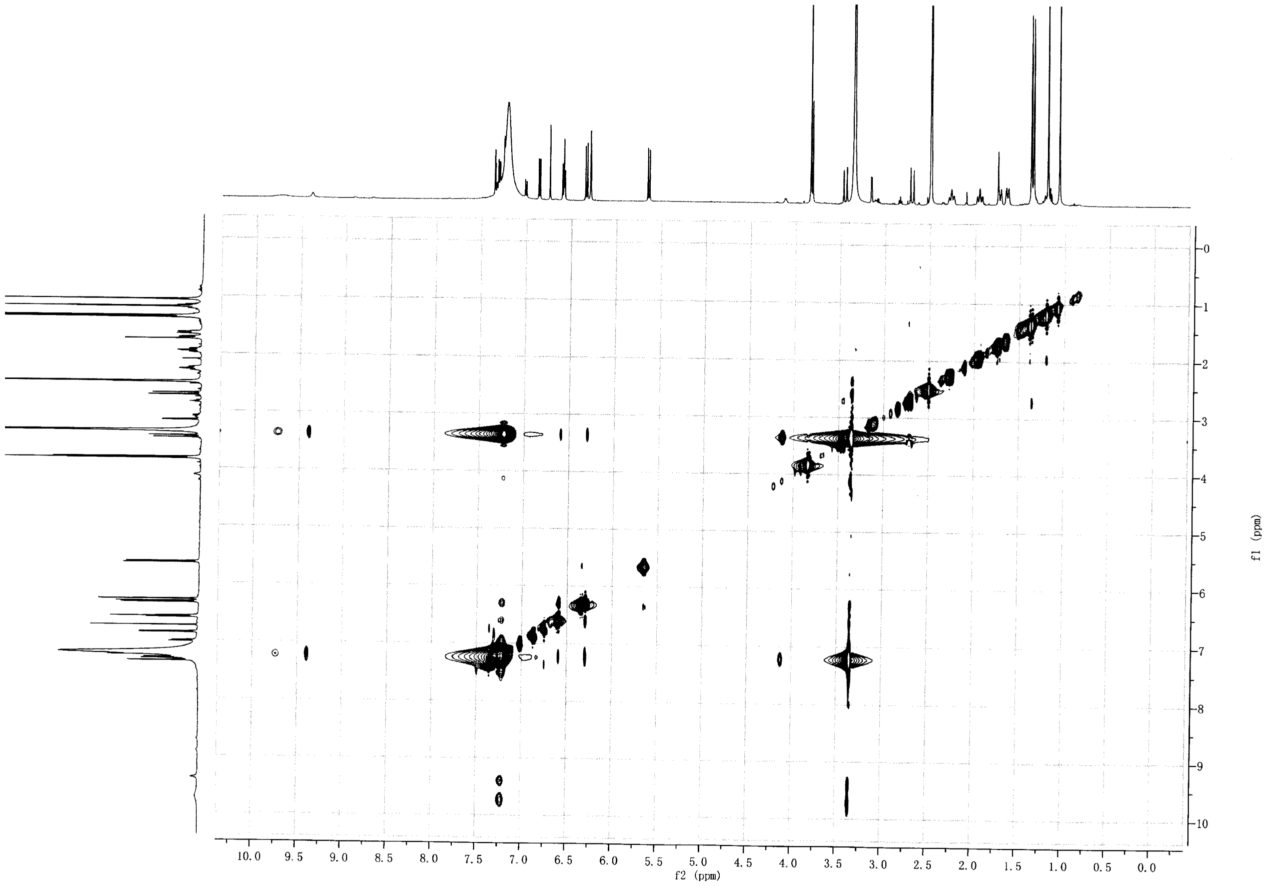

Figure S17. HR-ESIMS spectrum of **2**.

Mass Spectrum SmartFormula Report

Analysis Info

Analysis Name	D:\Data\MS\data\201404\nongxuhua-NQ-25_pos.d	Acquisition Date	4/18/2014 4:41:56 PM
Method	POS_100-2000_Dirrect Infusion.m	Operator	SCSIO
Sample Name	SCSIO	Instrument / Ser#	maXis 29
Comment			

Acquisition Parameter

Source Type	ESI	Ion Polarity	Positive	Set Nebulizer	0.4 Bar
Focus	Not active	Set Capillary	4500 V	Set Dry Heater	180 °C
Scan Begin	100 m/z	Set End Plate Offset	-500 V	Set Dry Gas	4.0 l/min
Scan End	2000 m/z	Set Collision Cell RF	2000.0 Vpp	Set Divert Valve	Waste

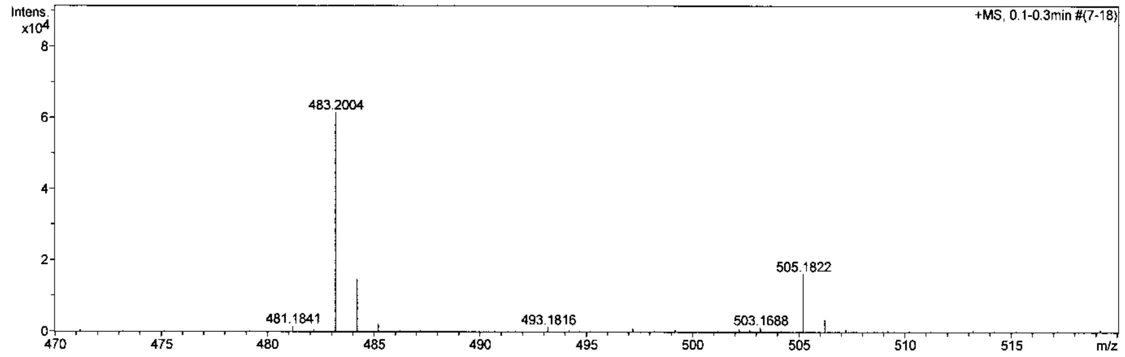

Meas. m/z	#	Formula	Score	m/z	err [mDa]	err [ppm]	mSigma	rdb	e⁻ Conf	N-Rule
483.2004	1	C 23 H 27 N 6 O 6	63.15	483.1987	-1.8	-3.7	20.5	13.5	even	ok
	2	C 27 H 31 O 8	83.91	483.2013	0.9	1.9	31.4	12.5	even	ok
	3	C 24 H 23 N 10 O 2	100.00	483.2000	-0.4	-0.9	34.5	18.5	even	ok
	4	C 28 H 27 N 4 O 4	25.84	483.2027	2.3	4.7	41.4	17.5	even	ok
505.1822	1	C 23 H 26 N 6 Na O 6	71.29	505.1806	-1.6	-3.2	37.0	13.5	even	ok
	2	C 27 H 30 Na O 8	83.88	505.1833	1.1	2.1	43.5	12.5	even	ok
	3	C 24 H 22 N 10 Na O 2	100.00	505.1819	-0.3	-0.5	51.4	18.5	even	ok
	4	C 28 H 26 N 4 Na O 4	22.22	505.1846	2.4	4.8	55.9	17.5	even	ok

Bruker Compass DataAnalysis 4.0 printed: 4/18/2014 4:54:11 PM Page 1 of 1

Figure S18. IR spectrum of **2**.

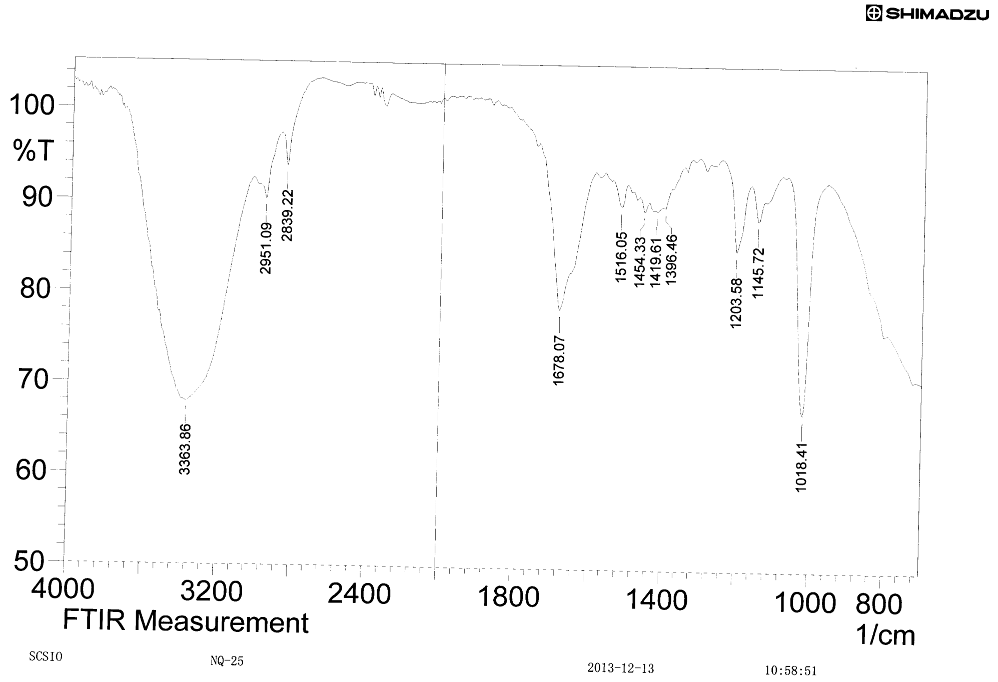

Figure S19. ^{1}H NMR spectrum of 3.

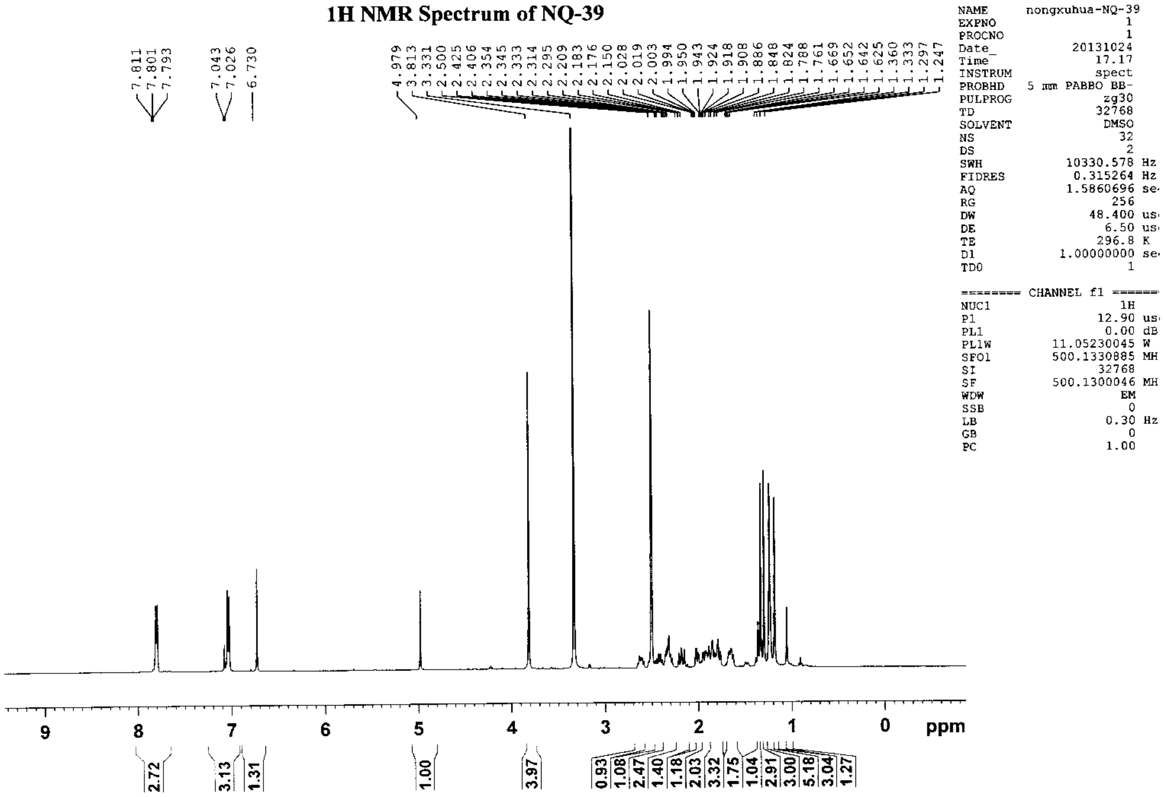

Figure S20. ^{13}C NMR and *DEPT135* spectrum of **3**.

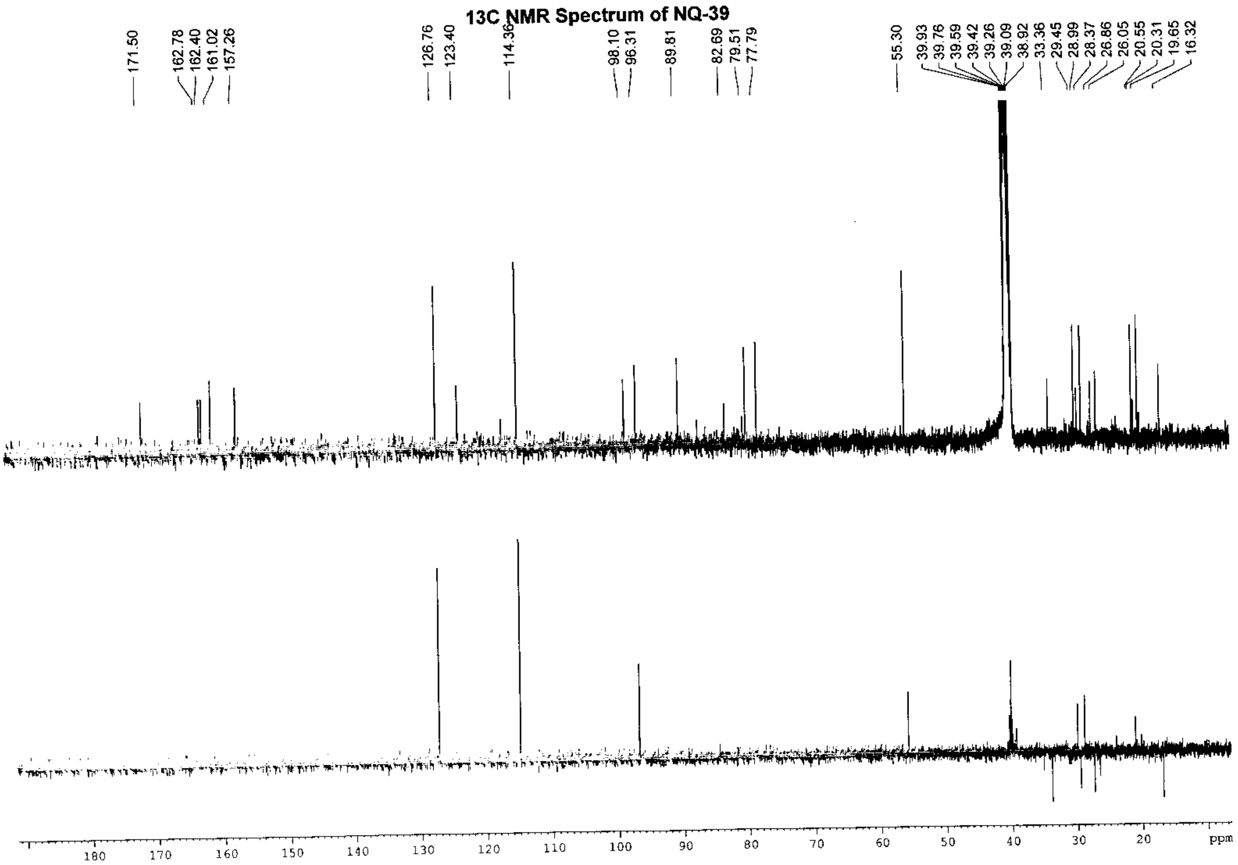

Figure S21. HSQC spectrum of **3**.

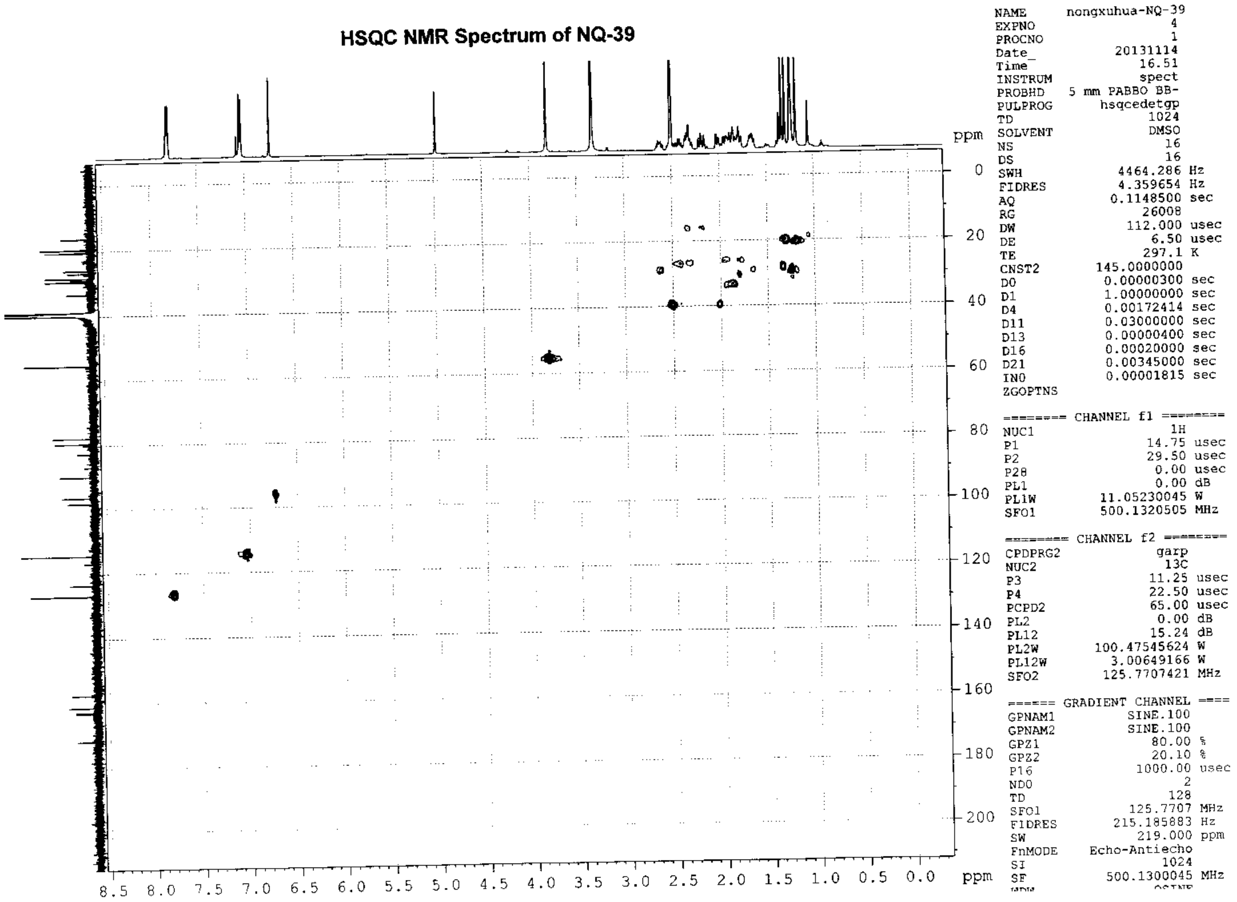

Figure S22. HMBC spectrum of **3**.

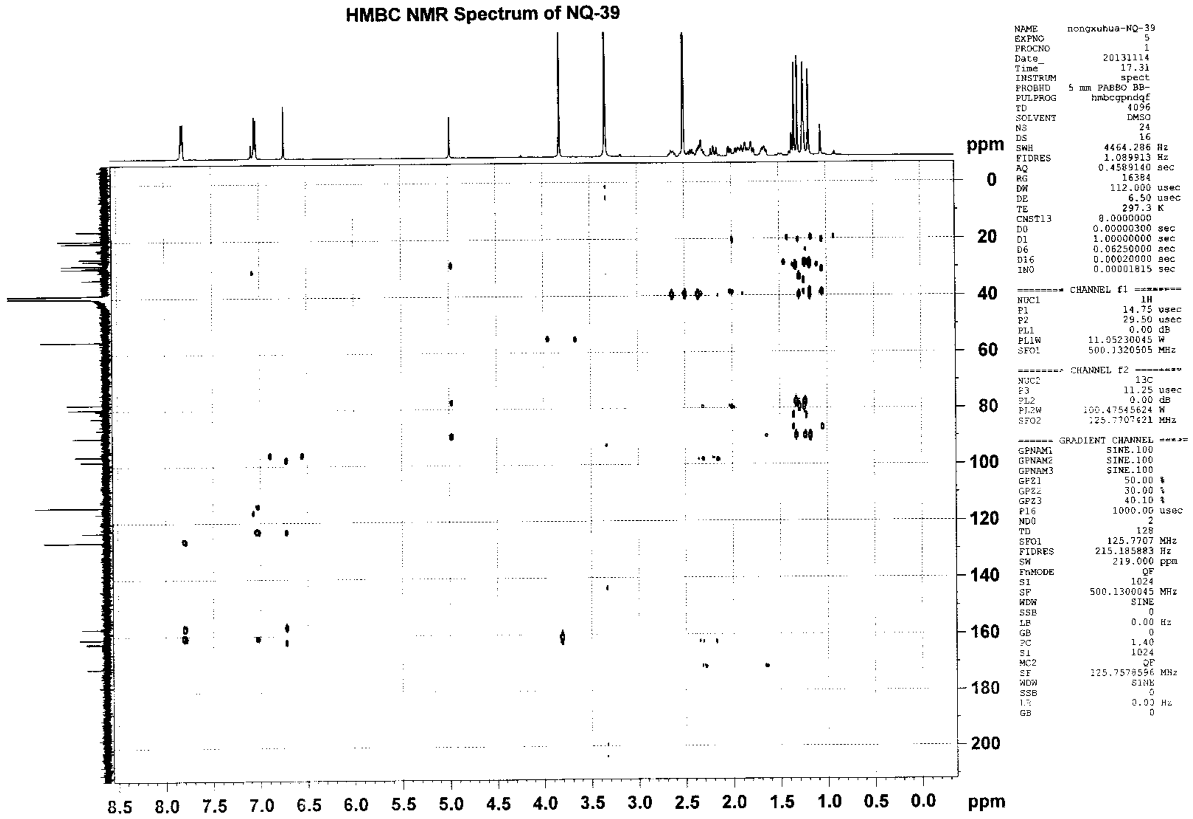

Figure S23. ^1H-^1H COSY spectrum of **3**.

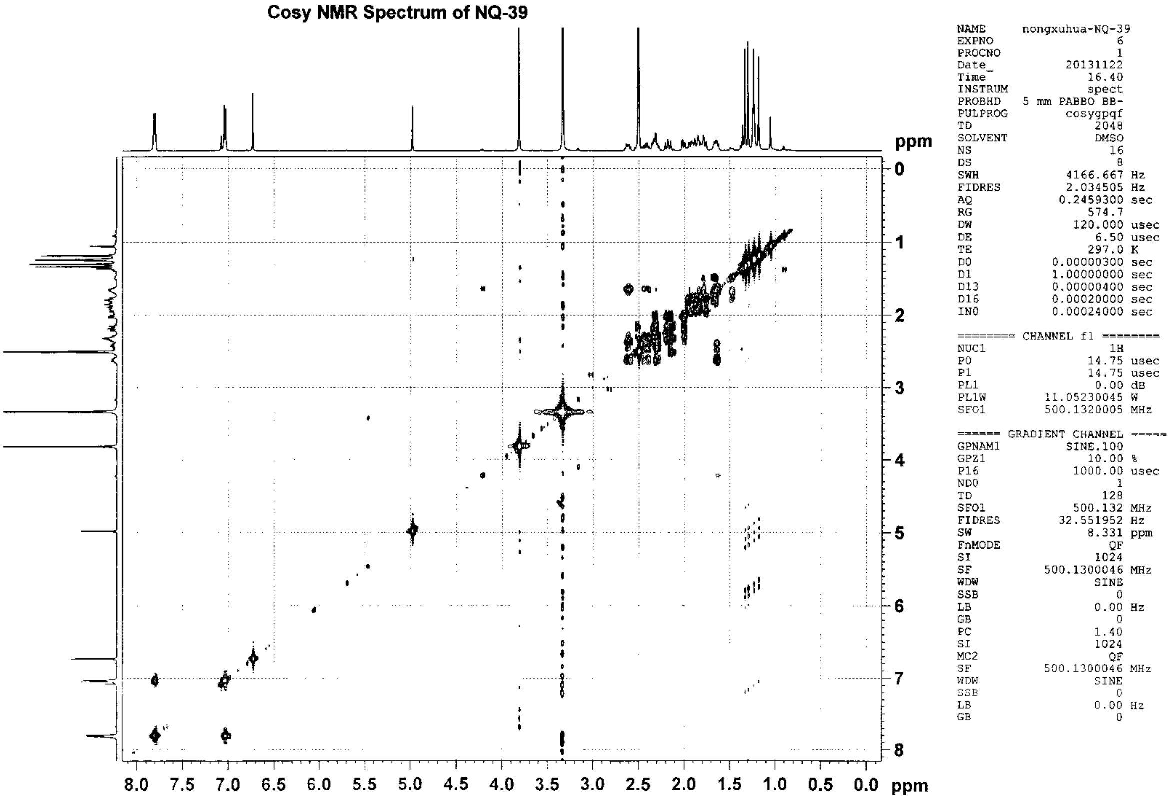

Figure S24. NOESY spectrum of **3**.

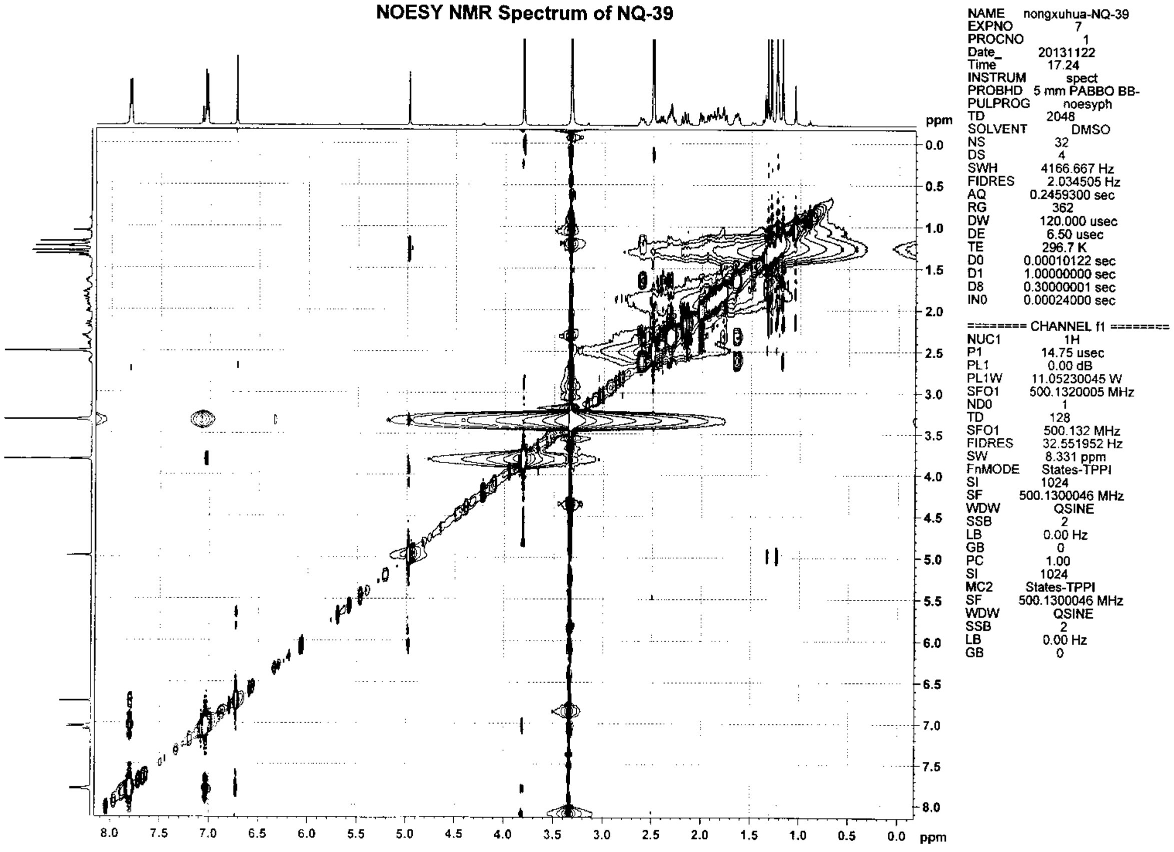

Figure S25. HR-ESIMS spectrum of **3**.

Mass Spectrum SmartFormula Report

Analysis Info

Analysis Name	D:\Data\MS\data\201311\nongxuhua_NQ-39_pos.d	Acquisition Date	11/26/2013 3:11:35 PM
Method	POS_100-2000_Dirrect Infusion.m	Operator	SCSIO
Sample Name	SCSIO	Instrument / Ser#	maXis 29
Comment			

Acquisition Parameter

Source Type	ESI	Ion Polarity	Positive	Set Nebulizer	0.3 Bar
Focus	Not active	Set Capillary	4500 V	Set Dry Heater	180 °C
Scan Begin	100 m/z	Set End Plate Offset	-500 V	Set Dry Gas	4.0 l/min
Scan End	1500 m/z	Set Collision Cell RF	2000.0 Vpp	Set Divert Valve	Waste

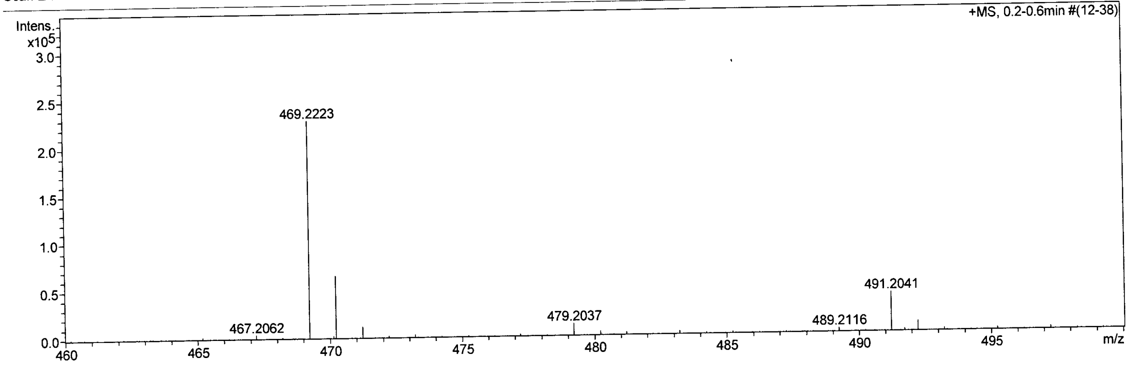

Meas. m/z	#	Formula	Score	m/z	err [mDa]	err [ppm]	mSigma	rdb	e⁻ Conf	N-Rule
469.2223	1	C 27 H 33 O 7	100.00	469.2221	-0.2	-0.5	6.2	11.5	even	ok
491.2041	1	C 27 H 32 Na O 7	100.00	491.2040	-0.0	-0.1	23.5	11.5	even	ok
937.4375	1	C 54 H 65 O 14	100.00	937.4369	-0.6	-0.7	22.4	22.5	even	ok
959.4191	1	C 54 H 64 Na O 14	100.00	959.4188	-0.3	-0.3	33.8	22.5	even	ok

Bruker Compass DataAnalysis 4.0 printed: 11/26/2013 3:43:17 PM Page 1 of 1

Figure S26. IR spectrum of 3.

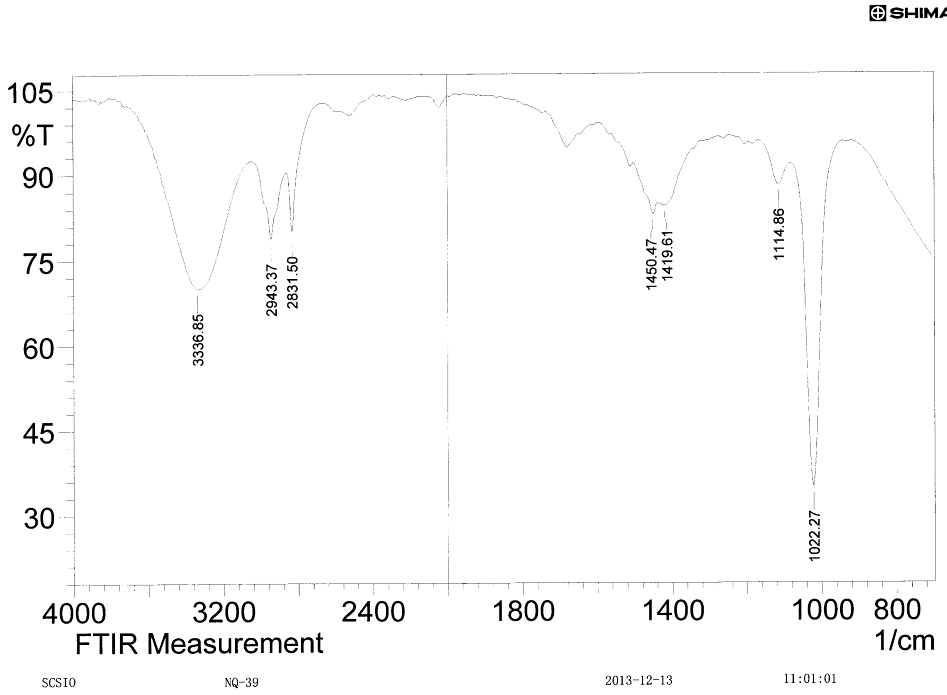

Figure S27. ^{1}H NMR spectrum of **9**.

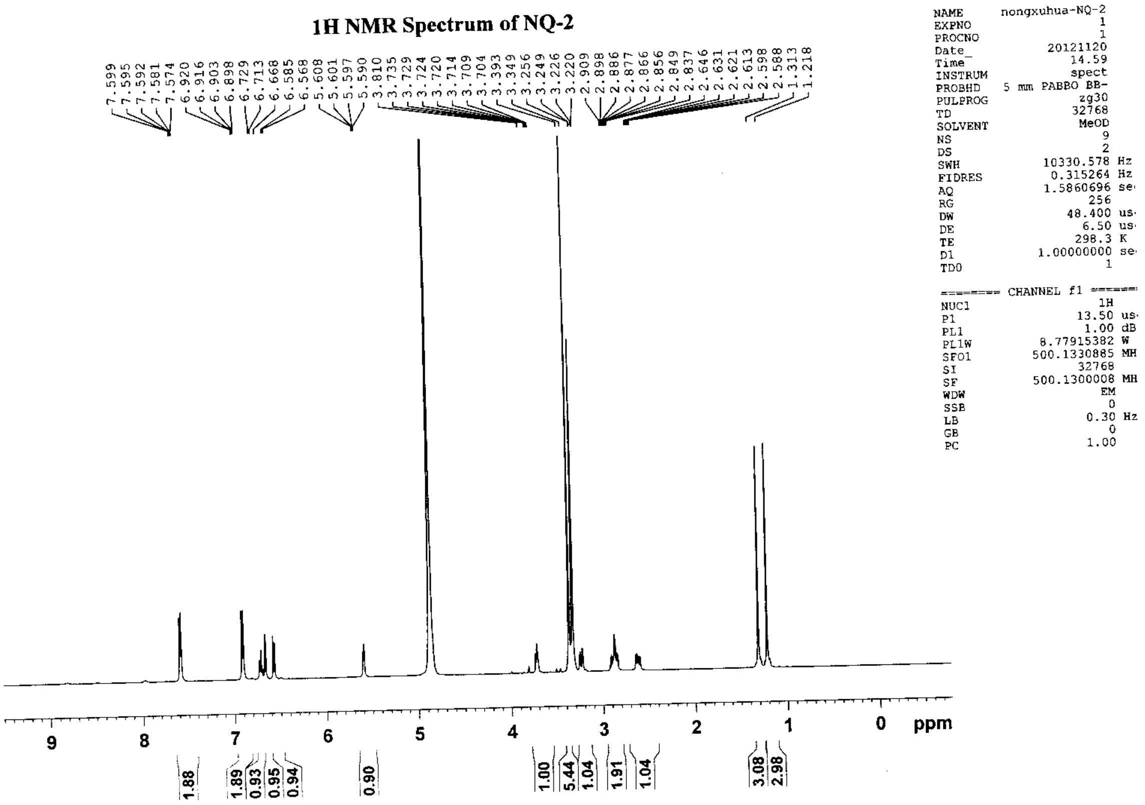

Figure S28. ^{13}C NMR spectrum of **9**.

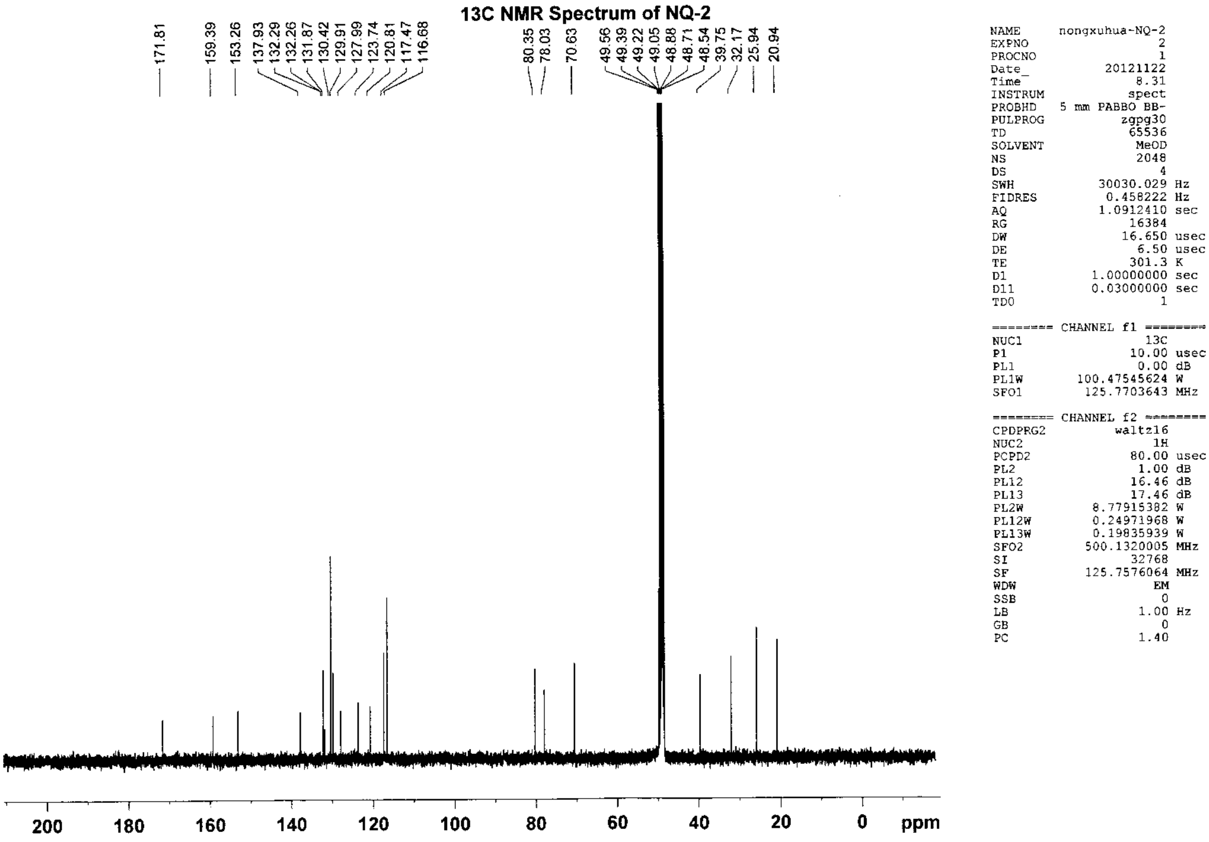

Figure S29. HSQC spectrum of **9**.

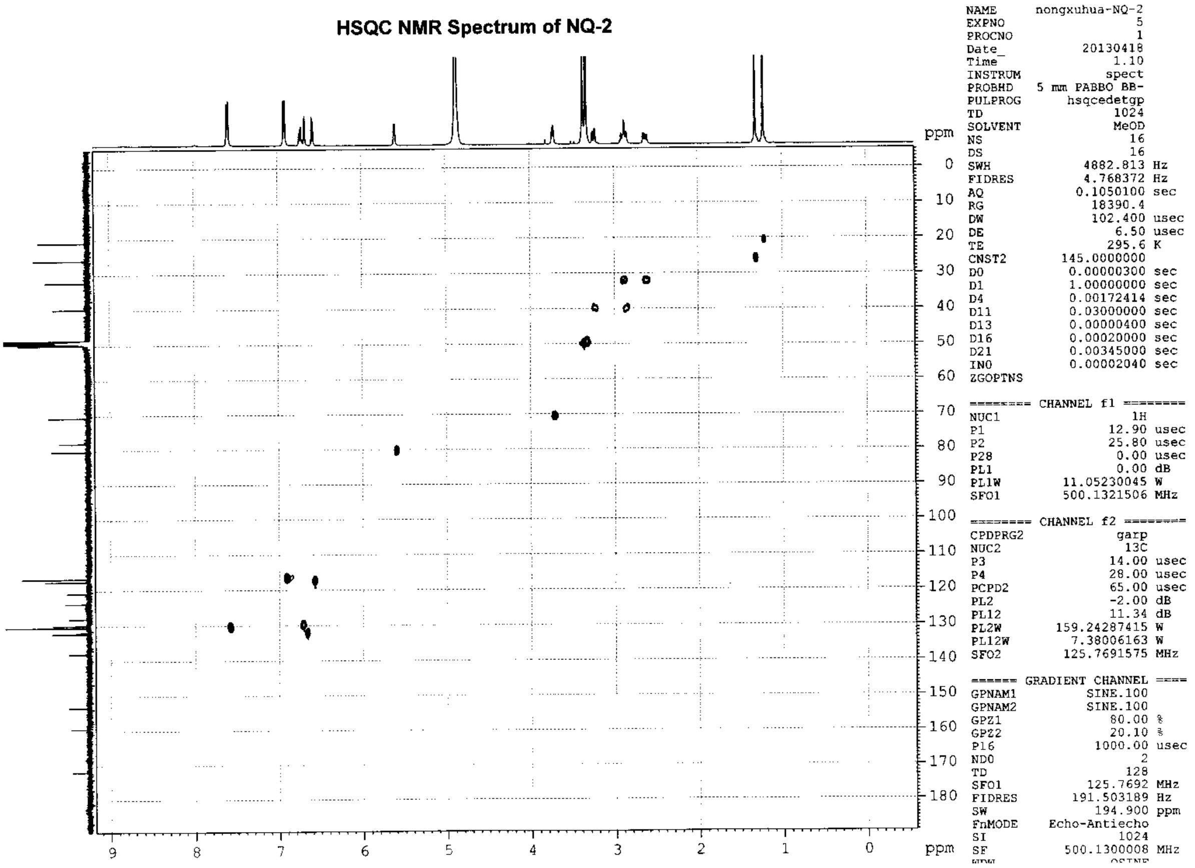

Figure S30. HR-ESIMS spectrum of **9**.

Mass Spectrum SmartFormula Report

Analysis Info

Analysis Name	D:\Data\MS\data\201304\nongxuhua-NQ-2 _pos.d	Acquisition Date	4/27/2013 4:24:50 PM
Method	POS_100-2000_Dirrect Infusion.m	Operator	SCSIO
Sample Name	SCSIO	Instrument / Ser#	maXis 29
Comment			

Acquisition Parameter

Source Type	ESI	Ion Polarity	Positive	Set Nebulizer	0.3 Bar
Focus	Not active	Set Capillary	3800 V	Set Dry Heater	180 °C
Scan Begin	100 m/z	Set End Plate Offset	-500 V	Set Dry Gas	4.0 l/min
Scan End	2000 m/z	Set Collision Cell RF	2000.0 Vpp	Set Divert Valve	Waste

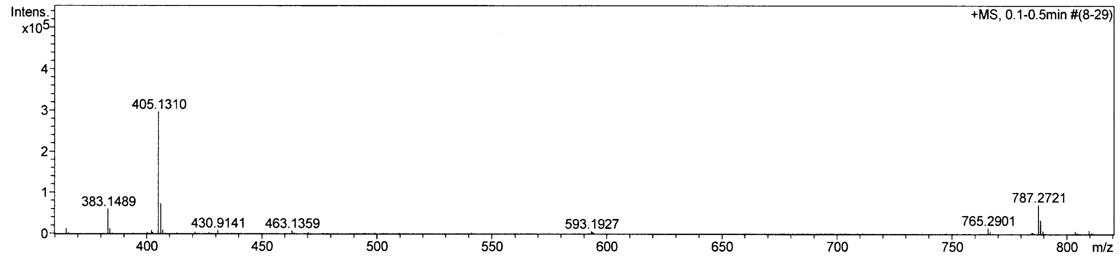

Meas. m/z	#	Formula	Score	m/z	err [mDa]	err [ppm]	mSigma	rdb	e⁻ Conf	N-Rule
383.1489	1	C 22 H 23 O 6	100.00	383.1489	-0.0	-0.0	29.0	11.5	even	ok
	2	C 19 H 15 N 10	48.90	383.1476	-1.3	-3.5	29.9	17.5	even	ok
	3	C 23 H 19 N 4 O 2	35.06	383.1503	1.3	3.5	42.9	16.5	even	ok
405.1310	1	C 22 H 21 N 2 Na 4	38.44	405.1290	-1.9	-4.8	1.2	11.5	even	ok
	2	C 19 H 14 N 10 Na	53.29	405.1295	-1.4	-3.6	3.0	17.5	even	ok
	3	C 21 H 19 N 4 Na 2 O 2	63.01	405.1298	-1.2	-2.9	3.5	13.5	even	ok
	4	C 22 H 22 Na O 6	100.00	405.1309	-0.1	-0.2	7.9	11.5	even	ok
	5	C 23 H 18 N 4 Na O 2	52.81	405.1322	1.2	3.1	10.4	16.5	even	ok
	6	C 24 H 20 N 2 Na 3	79.21	405.1314	0.5	1.1	11.7	14.5	even	ok
765.2901	1	C 39 H 45 N 2 O 14	16.83	765.2865	-3.6	-4.7	28.4	18.5	even	ok
	2	C 40 H 41 N 6 O 10	46.57	765.2879	-2.3	-2.9	38.5	23.5	even	ok
	3	C 38 H 29 N 20	37.74	765.2879	-2.3	-3.0	45.7	34.5	even	ok
	4	C 41 H 37 N 10 O 6	87.98	765.2892	-0.9	-1.2	48.2	28.5	even	ok
	5	C 44 H 45 O 12	100.00	765.2906	0.4	0.6	52.1	22.5	even	ok
	6	C 42 H 33 N 14 O 2	76.56	765.2905	0.4	0.6	60.4	33.5	even	ok
	7	C 45 H 41 N 4 O 8	33.46	765.2919	1.8	2.3	61.4	27.5	even	ok

Bruker Compass DataAnalysis 4.0 printed: 4/27/2013 4:33:00 PM Page 1 of 2

Figure S31. IR spectrum of **9**.

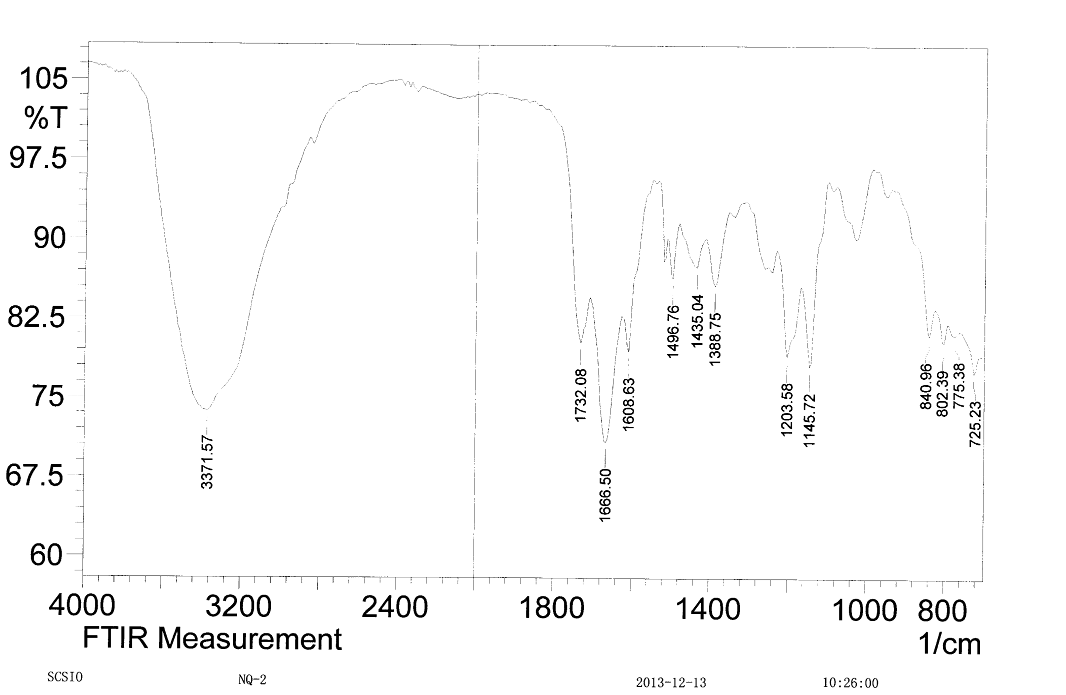

Figure S32. ^{1}H-NMR spectrum of 10.

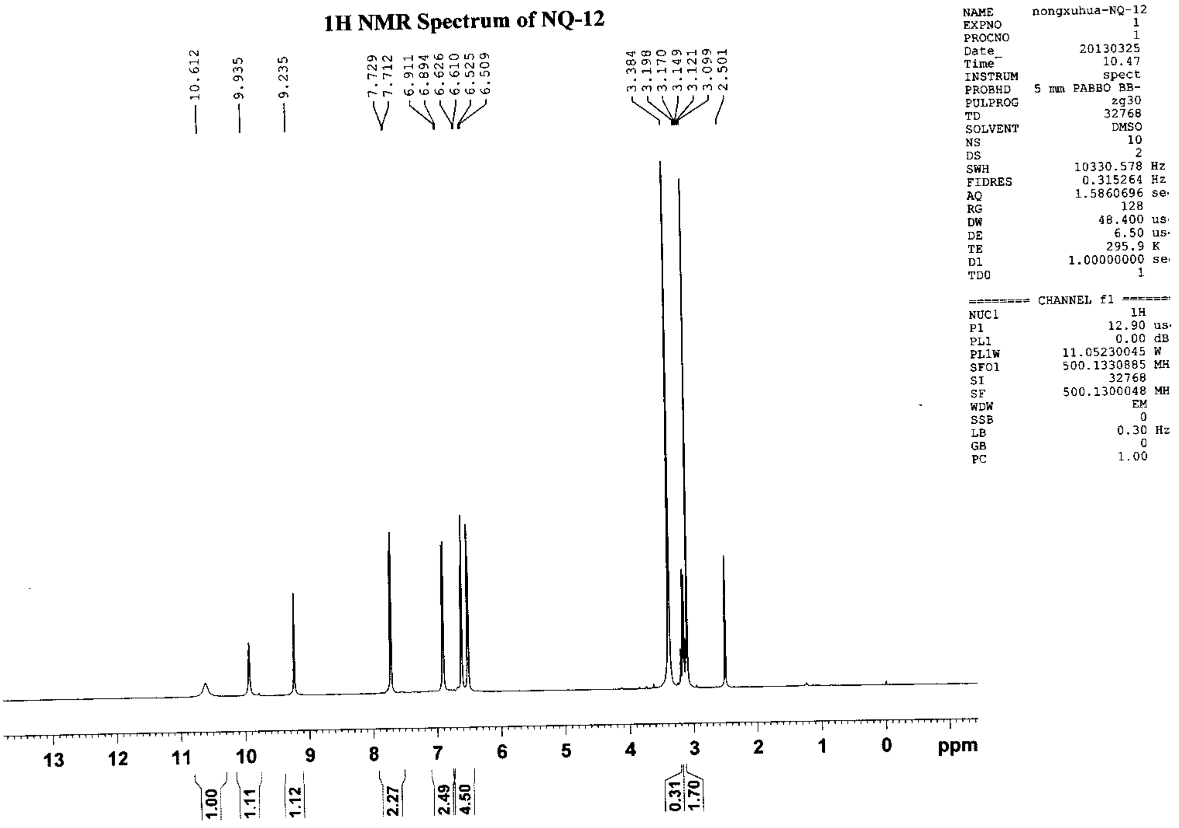

Figure S33. ^{13}C NMR and *DEPT135* spectra of **10**.

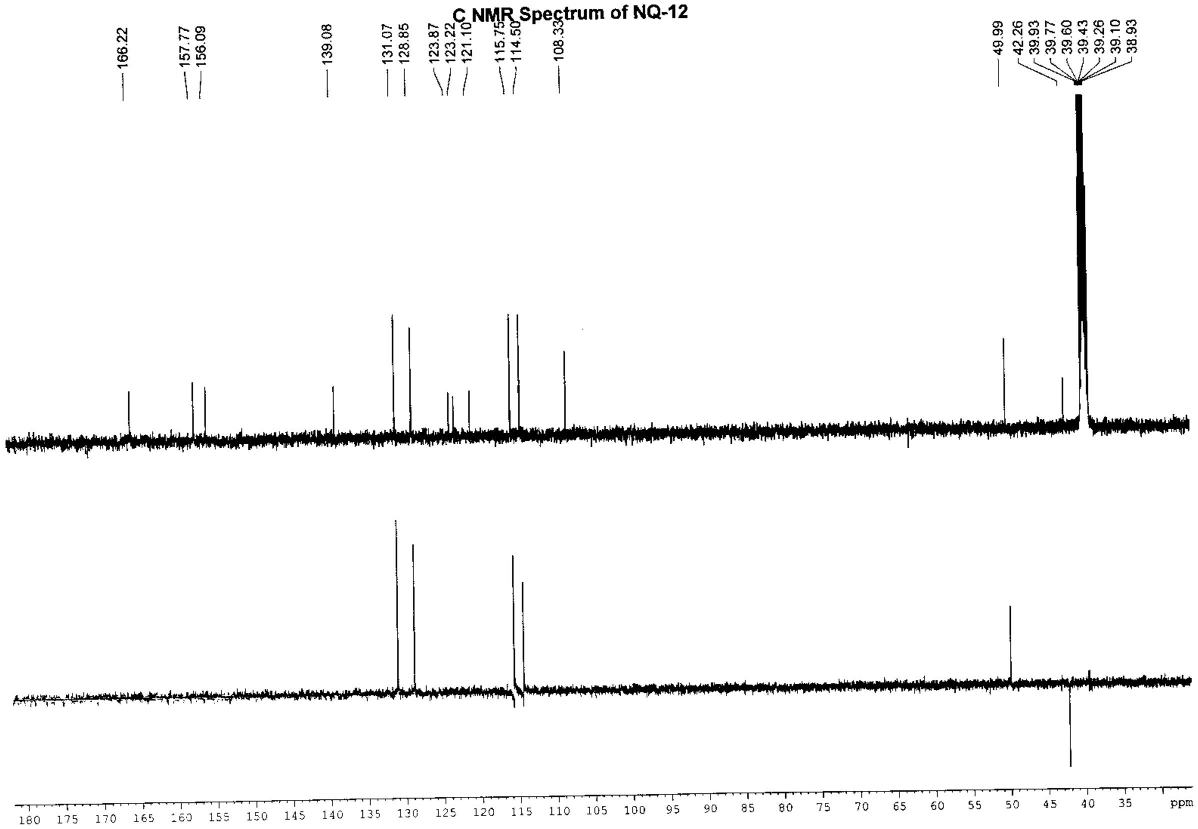

Figure S34. HSQC spectrum of **10**.

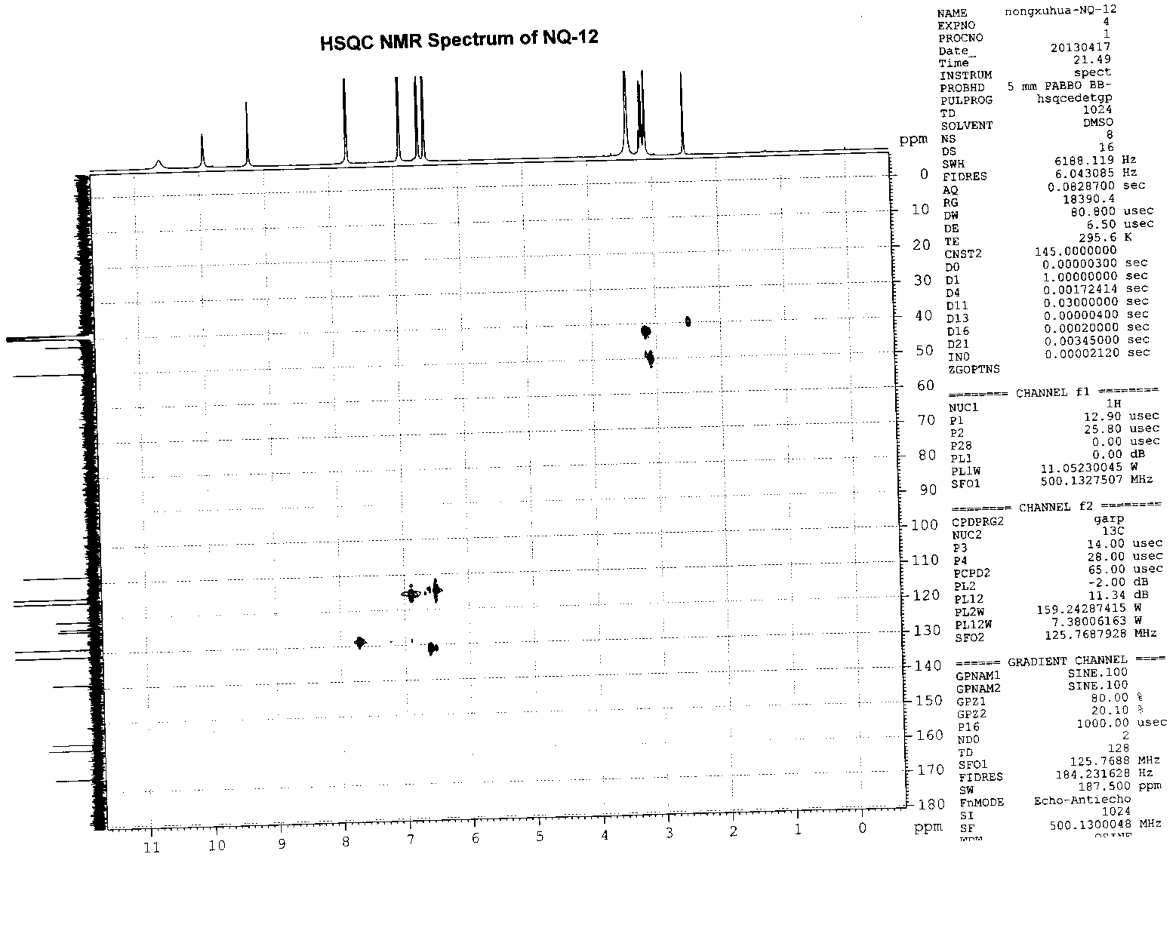

Figure S35. HMBC spectrum of **10**.

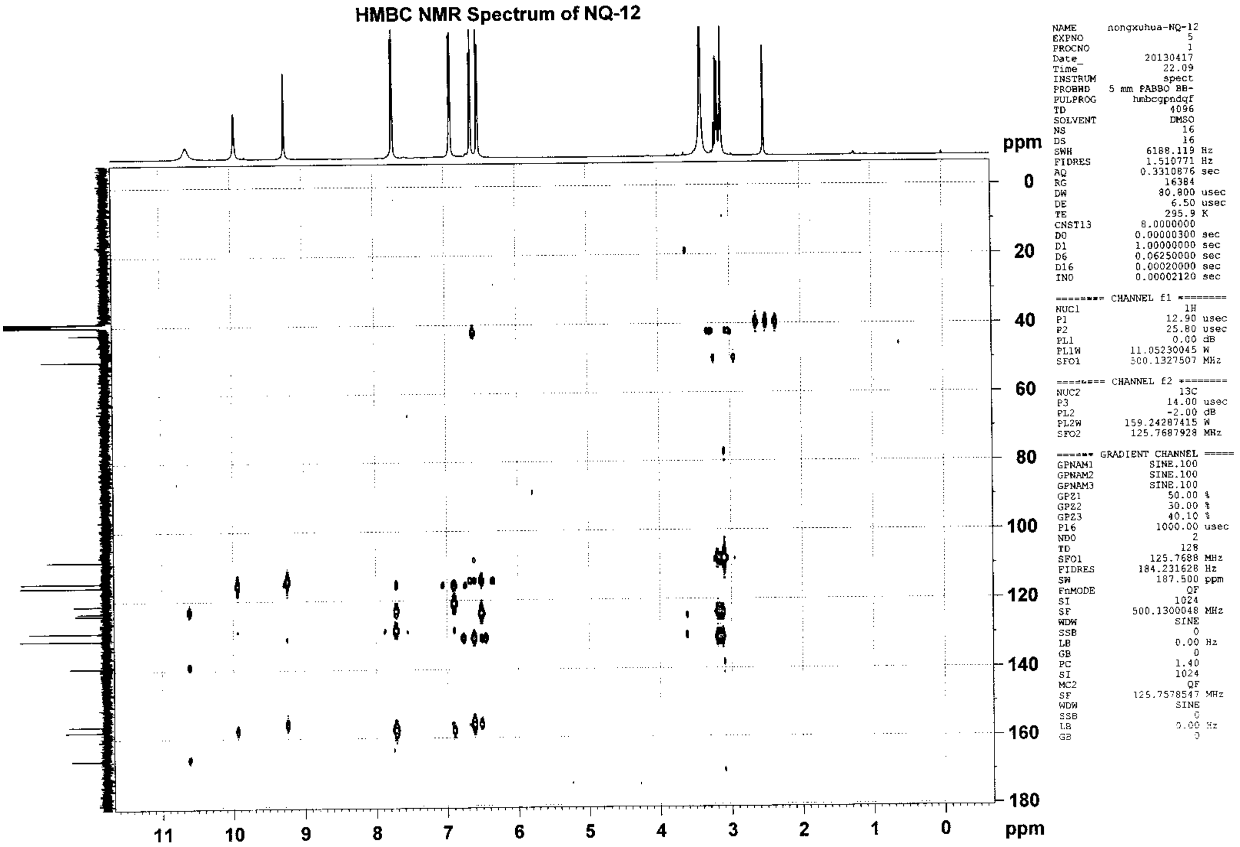

Figure S36. ^{1}H-^{1}H COSY spectrum of **10**.

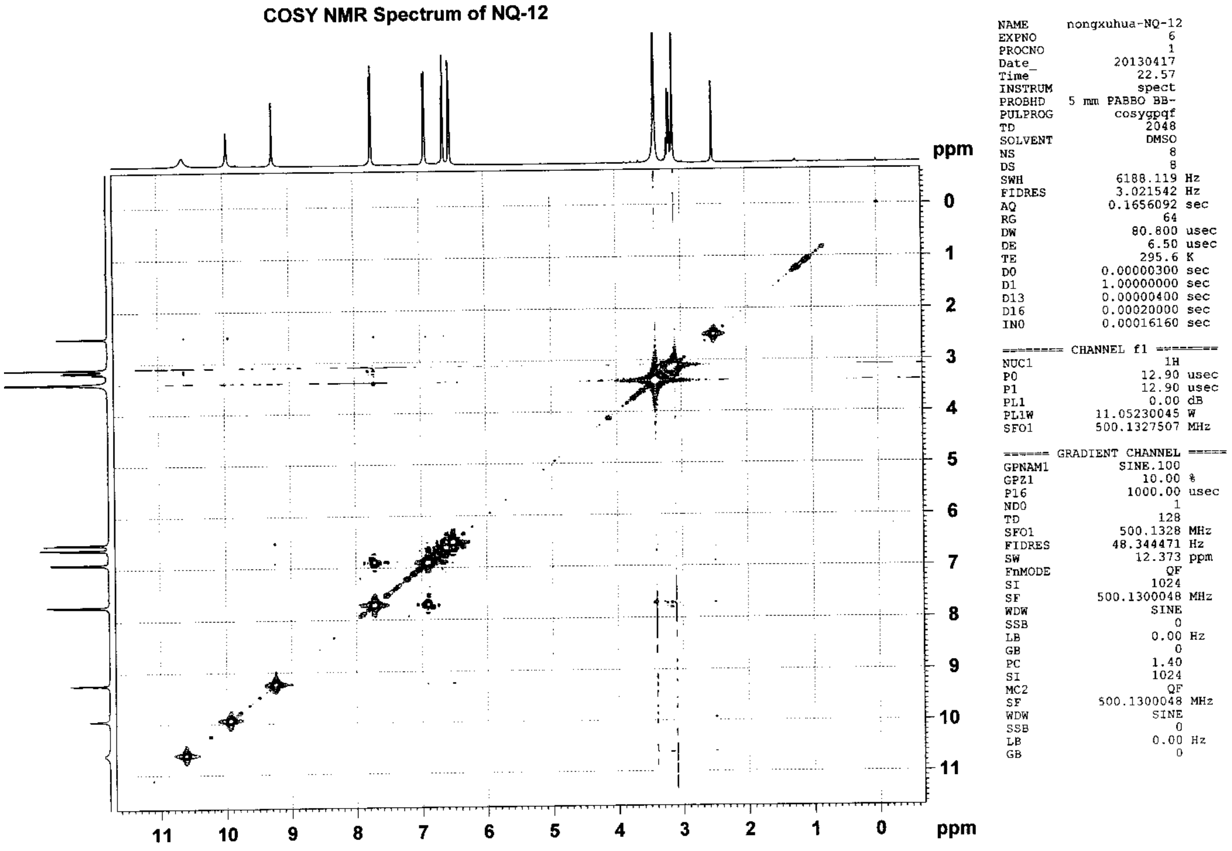

Figure S37. HR-ESIMS spectrum of **10**.

Mass Spectrum SmartFormula Report

Analysis Info

Analysis Name	D:\Data\MS\data\201304\nongxuhua-NQ-12 pos.d	Acquisition Date	4/11/2013 10:00:17 AM
Method	POS_100-2000_Dirrect Infusion.m	Operator	SCSIO
Sample Name	SCSIO	Instrument / Ser#	maXis 29
Comment			

Acquisition Parameter

Source Type	ESI	Ion Polarity	Positive	Set Nebulizer	0.3 Bar
Focus	Not active	Set Capillary	3800 V	Set Dry Heater	180 °C
Scan Begin	100 m/z	Set End Plate Offset	-500 V	Set Dry Gas	4.0 l/min
Scan End	2000 m/z	Set Collision Cell RF	2000.0 Vpp	Set Divert Valve	Waste

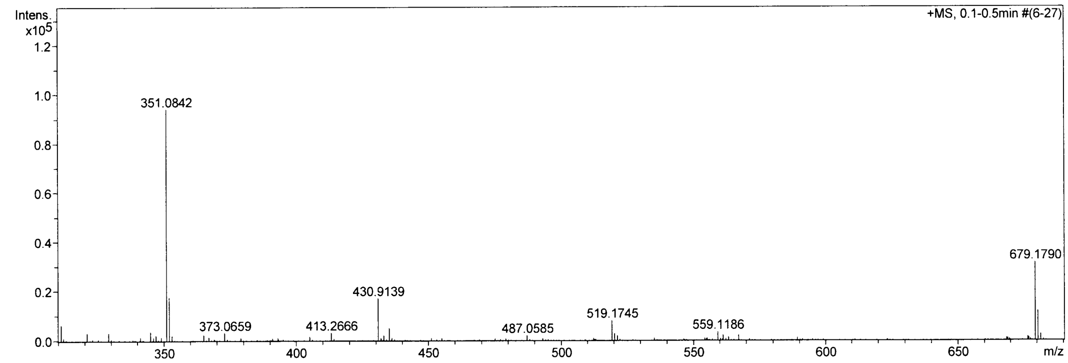

Meas. m/z	#	Formula	Score	m/z	err [mDa]	err [ppm]	mSigma	rdb	e⁻ Conf	N-Rule
329.1018	1	C 18 H 17 O 6	100.00	329.1020	0.1	0.4	5.5	10.5	even	ok
	2	C 15 H 9 N 10	50.75	329.1006	-1.2	-3.7	11.3	16.5	even	ok
	3	C 19 H 13 N 4 O 2	42.75	329.1033	1.5	4.4	13.0	15.5	even	ok
351.0842	1	C 18 H 16 Na O 6	100.00	351.0839	-0.3	-0.7	6.0	10.5	even	ok
	2	C 15 H 8 N 10 Na	45.70	351.0826	-1.6	-4.6	7.6	16.5	even	ok
	3	C 19 H 12 N 4 Na O 2	51.04	351.0852	1.1	3.1	19.3	15.5	even	ok
679.1790	1	C 32 H 28 N 6 Na O 10	17.42	679.1759	-3.1	-4.6	4.4	21.5	even	ok
	2	C 33 H 24 N 10 Na O 6	50.32	679.1772	-1.8	-2.6	9.0	26.5	even	ok
	3	C 30 H 16 N 20 Na	15.18	679.1759	-3.1	-4.6	11.5	32.5	even	ok

Bruker Compass DataAnalysis 4.0 printed: 4/11/2013 10:04:37 AM Page 1 of 2

Figure S38. IR spectrum of **10**.

SHIMADZU

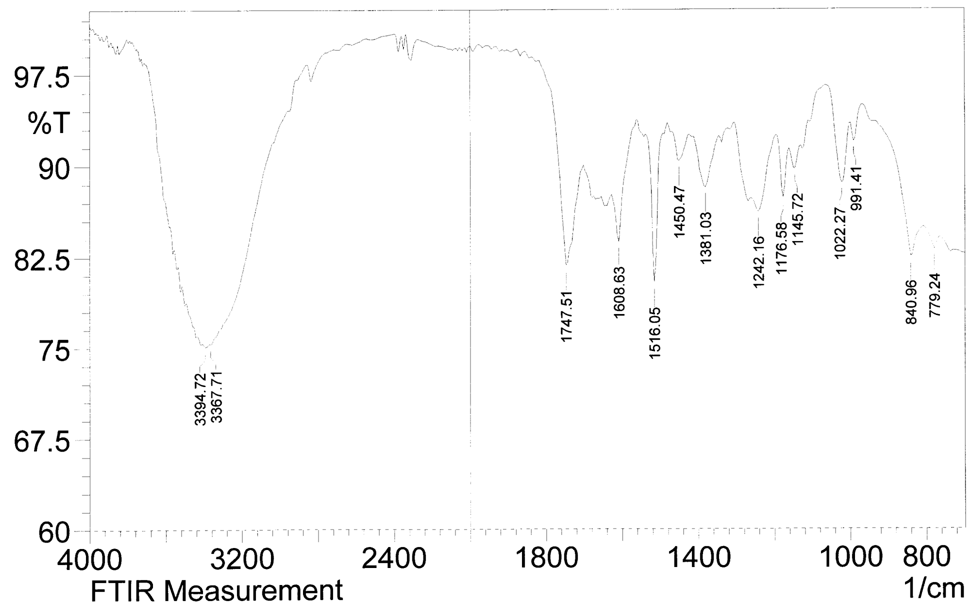

SCSIO NQ-12 2013-12-13 11:17:24

Figure S39. Comparison of the experimental CD spectra of 9, 11, 12 and 4-(4-hydroxyphenyl)-5-(4-hydroxyphenylmethyl)-2-hydroxyfurane-2-one (18).

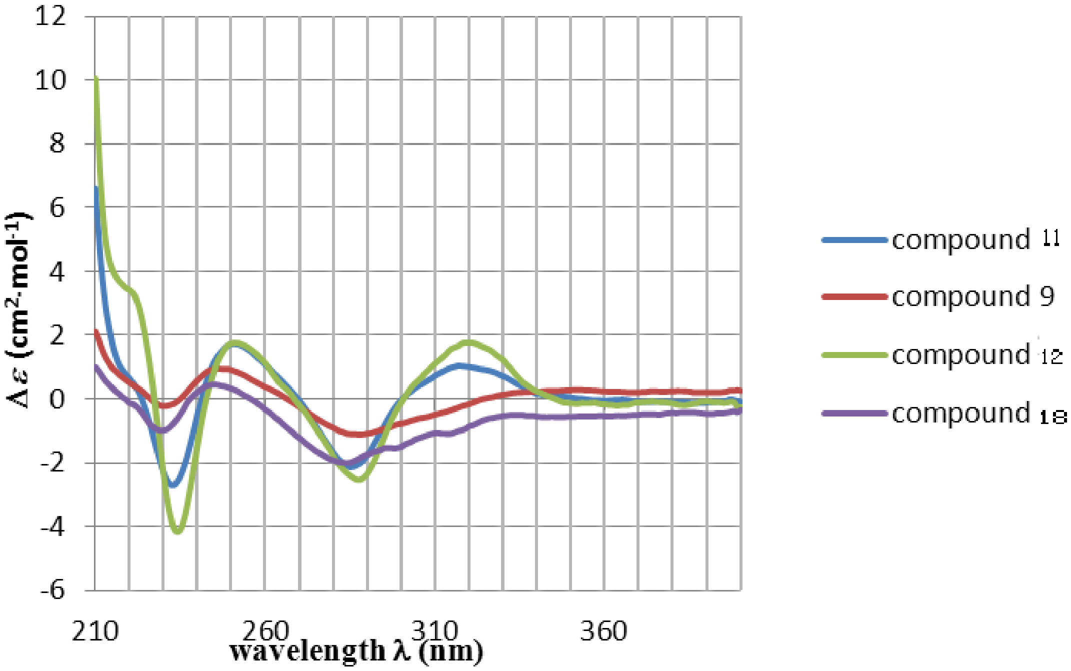

S1. Isolation and Purification of Compounds 1–17

The extract was subjected to silica gel CC using gradient elution with a $CHCl_3/CH_3OH$ solvent system at the ratios of 100:0, 98:2, 95:5, 90:10, 80:20, 50:50, and 0:100 (v/v) to give eight fractions (Fr.1–Fr.8). Fr.1 (3.9 g) was subjected to a silica gel CC eluting with $CHCl_3/CH_3CO\ CH_3$ at the ratios of 90:10, 80:20, 70:30, 50:50, 100:0 (v:v), to offer Fr.1-1~8. Subfraction Fr.1-2 was isolated by MPLC with an ODS column, eluting with CH_3CN-H_2O (from 10:90 to 100:0, 90 min, 20 mL/min) to give Fr.1-2-1~6. Subfraction Fr.1-2-5 was purified by SP-RP HPLC, eluting with CH_3CN/H_2O (37:53), to obtain **13** (t_R = 55 min, 15 mg). Subfraction Fr.1-2-6 was purified by SP-RP HPLC, eluting with CH_3CN/H_2O (65:35), to obtain **6** (t_R = 77 min, 2 mg). Subfraction Fr.1-3 was purified by SP-RP HPLC, eluting with CH_3OH/H_2O (60:40), to obtain **12** (t_R = 70 min, 15 mg), **4** (t_R = 53 min, 15 mg). Subfraction Fr.1-5 was purified by SP-RP HPLC, eluting with CH_3OH/H_2O (55:45), to obtain **17** (t_R = 30 min, 3 mg), **7** (t_R = 60 min, 4 mg). Subfraction Fr.1-6 was isolated by MPLC with an ODS column, eluting with CH_3CN-H_2O (from 10:90 to 100: 0, 60 min, 20 mL/min) to give Fr.1-6-1~4. Fr.1-6-3 was purified by SP-RP HPLC, eluting with CH_3OH/H_2O (70:30), to obtain **8** (t_R = 33 min, 2 mg), **3** (t_R = 41 min, 4 mg). Subfraction Fr.1-7 was isolated by MPLC with an ODS column, eluting with CH_3CN-H_2O (from 10:90 to 100:0, 60 min, 20 mL/min) to give Fr.1-7-1~4. And subfraction Fr.1-7-2 was purified by SP-RP HPLC, eluting with CH_3CN/H_2O (45:55), to obtain **1** (t_R = 36 min, 3 mg), **5** (t_R = 45 min, 6 mg), **2** (t_R = 47 min, 4 mg). Fr.2 (11 g) was subjected to a silica gel CC eluting with $CHCl_3/CH_3COCH_3$ at the ratios of 90:10, 80:20, 70:30, 50:50, 100:0 (v:v), to offer Fr.2-1~6. Subfraction Fr.2-2 was seperated by MPLC with an ODS column, eluting with CH_3OH-H_2O (from 10:90 to 100:0, 60 min, 20 mL/min) to give Fr.2-2-1~3. And subfraction Fr.2-2-1 was purified by SP-RP HPLC, eluting with CH_3CN/H_2O (57:43), to obtain **14** (t_R = 30 min, 10 mg). Fr.4 (1.45 g) was isolated by MPLC with an ODS column, eluting with CH_3OH-H_2O (from 15:85 to 100:0, 90 min, 20 mL/min) to give Fr.4-1~5. Fr.4-5 was

purified by SP-RP HPLC, eluting with CH_3CN/H_2O (46:54), to afford **11** (t_R = 23 min, 30 mg) and **9** (t_R = 19.0 min, 4 mg). Fr.5 (1.2 g) was isolated by MPLC with an ODS column, eluting with CH_3OH-H_2O (from 15:85 to 100:0, 90min, 20 mL/min) to give Fr.5-1~5. Subfraction Fr.5-3 was purified by SP-RP HPLC, eluting with CH_3OH/H_2O (55:45), to obtain **10** (t_R = 39 min, 8 mg). Fr.7 (3 g) was subjected to Sephadex LH-20 CC eluting with CH_3OH to collect Fr.7-1~4. Further subfraction Fr.7-4 (1.8 g) was isolated by MPLC with an ODS column, eluting with CH_3OH-H_2O (from 15:85 to 100:0, 90 min, 20 mL/min) to give Fr.7-4-1~4. And then subfraction Fr.7-4-4 was purified by SP-RP HPLC, eluting with CH_3OH/H_2O (63:37), to obtain **15** (t_R = 37 min, 8 mg), **16** (t_R = 14 min, 6 mg).

S2. Structures of Compounds 1–17

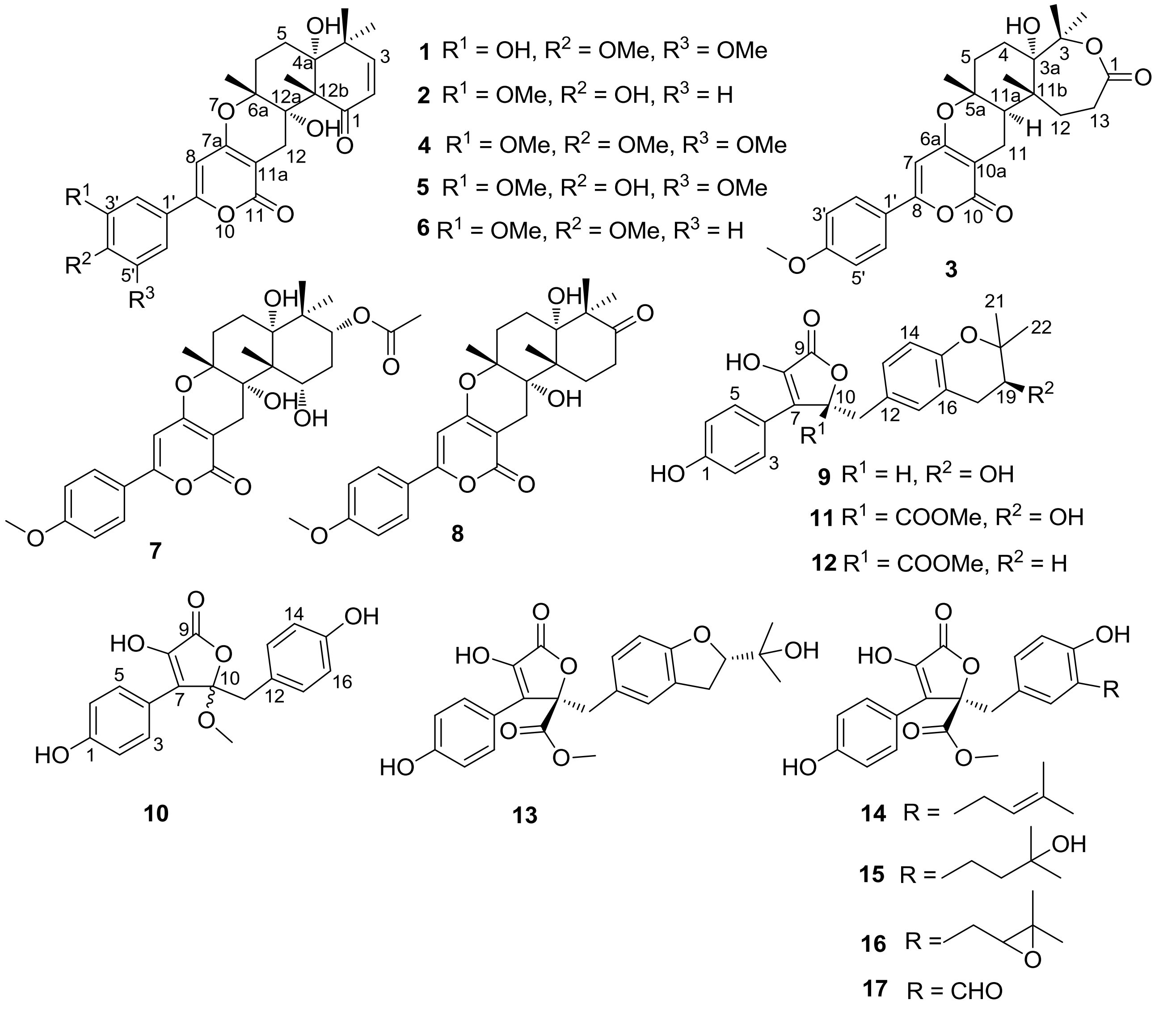

© 2014 by the authors; licensee MDPI, Basel, Switzerland. This article is an open access article distributed under the terms and conditions of the Creative Commons Attribution license (http://creativecommons.org/licenses/by/4.0/).
